# Clustering gene expression data with a penalized graph-based metric

**DOI:** 10.1186/1471-2105-12-2

**Published:** 2011-01-04

**Authors:** Ariel E Bayá, Pablo M Granitto

**Affiliations:** 1 CIFASIS French Argentine International Center for Information and Systems Sciences, UPCAM (France)/UNR-CONICET (Argentina), Bv 27 de Febrero 210 Bis, 2000 Rosario, República Argentina

## Abstract

**Background:**

The search for cluster structure in microarray datasets is a base problem for the so-called "-omic sciences". A difficult problem in clustering is how to handle data with a manifold structure, i.e. data that is not shaped in the form of compact clouds of points, forming arbitrary shapes or paths embedded in a high-dimensional space, as could be the case of some gene expression datasets.

**Results:**

In this work we introduce the Penalized k-Nearest-Neighbor-Graph (PKNNG) based metric, a new tool for evaluating distances in such cases. The new metric can be used in combination with most clustering algorithms. The PKNNG metric is based on a two-step procedure: first it constructs the k-Nearest-Neighbor-Graph of the dataset of interest using a low k-value and then it adds edges with a highly penalized weight for connecting the subgraphs produced by the first step. We discuss several possible schemes for connecting the different sub-graphs as well as penalization functions. We show clustering results on several public gene expression datasets and simulated artificial problems to evaluate the behavior of the new metric.

**Conclusions:**

In all cases the PKNNG metric shows promising clustering results. The use of the PKNNG metric can improve the performance of commonly used pairwise-distance based clustering methods, to the level of more advanced algorithms. A great advantage of the new procedure is that researchers do not need to learn a new method, they can simply compute distances with the PKNNG metric and then, for example, use hierarchical clustering to produce an accurate and highly interpretable dendrogram of their high-dimensional data.

## Background

The introduction of microarrays and other high-throughput technologies over the last years has changed fundamentally the biological and biomedical research. DNA microarrays, in particular, allow the simultaneous monitoring of thousands of genes on different experimental conditions or time points. Several problems can be faced with this technology. For example, it can be used for the identification of differentially expressed genes [1], which could highlight possible gene targets for more detailed molecular studies or drug treatments. Another application is to assign samples to known classes (class prediction) [2], using genetic profiles to improve, for example, the diagnosis of cancer patients. Both are supervised applications, in which all samples belong to a previously known class. It is very common, also, to use microarray data for (non-supervised) exploratory analysis. A common application is to use a clustering algorithm to group the different genes. As stated by Richards et al. [3], clustering in this way can help summarizing datasets, reducing from thousands of genes to a small number of clusters. It can also help find systemic effects, as looking for small changes in expression levels across many genes in a cluster could be a better strategy for finding the causes of complex, polygenic disorders than looking for large changes in single genes [4]. Clustering genes can be seen as a "classical" clustering problem, with thousands of examples (genes) measured over a small number of variables (tissues, experimental conditions, etc.). The other common non-supervised analysis is to cluster the samples (tissues/diseases/patients). The goal in this second situation is to find groups of samples sharing similar gene expression patterns. Seminal works in this area are Golub et al. [2] and Alizadeh et al. [5], both aimed at the discovery of new cancer subtypes. However, clustering samples is very different to clustering genes. In this case the analysis is made over so-called "wide datasets", with a few samples measured over thousands of features (genes). Dealing with high dimensional spaces is a known challenge for clustering procedures, as they usually fail to handle manifold-structured data, i.e. data that form low-dimensional, arbitrary shapes or paths through a high-dimensional space.

Clustering is a key component of pattern analysis methods, aiming at finding hidden structures in a set of data. Informally, the objective is to organize the samples in a way that "similar" objects are grouped together. It is a very active field of research [6-9], with applications that cover diverse problems, from image segmentation in computer vision [10] to characterizing customer groups based on purchasing patterns [11] and, as mentioned, the analysis of microarray expression data [12].

The problem of finding clusters in a dataset can be divided into three stages: i) measuring the similarity of the objects under analysis, ii) grouping the objects according to these similarities, and iii) evaluating the "goodness" of the clustering solution. The last stage has received little attention until recent years, when a growing interest in the problem can be noticed [13-15]. The second stage (finding clusters efficiently given a set of similarities between objects) has been widely studied in the literature [16] and several clustering algorithms have been introduced. They are usually divided into hierarchical and partitional methods [17]. Hierarchical clustering (HC) algorithms find successive clusters using previously defined ones, in an agglomerative ("bottom-up") or divisive ("top-down") way [18]. The result of this process is a binary tree, called a dendrogram [19]. HC has been extensively applied to microarray data [2,5,20-22]. De Souto et al. [12] noted that more than 90% of published clustering applications to microarray data use HC. Partitional clustering algorithms find all the clusters simultaneously as a division of the data and do not impose a hierarchical structure. One of the most widely used approaches is the K-Means [23] algorithm (or its related version PAM [24]) that, starting from *k *(usually random) clusters, searches iteratively for a locally optimal solution of the clustering problem. Partitional methods have also been successfully applied to microarray data [25-28]. Recently, Frey and Dueck [8] proposed the innovative and computationally efficient Affinity Propagation (AP) algorithm. According to this method, each data point is viewed as a node in a network. Nodes exchange messages until a set of cluster-centers emerges as a solution. The algorithm shares characteristics with both hierarchical and partitioning methods. To the best of our knowledge, the work of Michele Leone and Weigt [29] is the only published application of AP to microarray data.

Several clustering methods targeted to microarray data have been proposed [30-36], but, as discussed by De Souto et al. [12], most researchers still rely on the traditional HC method with Pearson's correlation or Euclidean metric, mostly because of its ease of use and availability. According to this, instead of developing new algorithms appropriate for high-dimensional microarray problems, we propose here to obtain better solutions going back to stage one of clustering, i.e., by finding better ways to measure similarities between data-points. These improved distances can be used by any base clustering method to produce better clustering results. In some sense, our view shares the spirit of kernel methods [37], looking for solutions to new problems by using appropriate new metrics together with well-known pattern analysis algorithms.

In the last years, several methods for characterizing the non-linear manifold where a high-dimensional dataset may lie were developed, like ISOMAP [38], Locally Linear Embedding [39] or Laplacian Eigenmaps [40]. Basically, they all look for local neighborhood relations that can be used to produce low dimensional projections of the data at hand. In this paper we discuss a new strategy to evaluate similarities in manifold spaces that easily extends the application of any clustering algorithm to these cases. Following ISOMAP, we first create the k-nearest-neighbor-graph (knn-graph) of the data, using a low k-value. If the graph is disconnected, which is expected in clustering problems, we add a number of edges (following different strategies that will be discussed later) in order to create a connected graph. The key point of our method is that the added edges have a highly penalized length. We then apply an appropriate algorithm to measure inter-point distances along the connected graph and use these measures as (dis)similarities. We call the method the PKNNG metric (for Penalized K-Nearest-Neighbor-Graph based metric). The PKNNG metric can be applied to any base measure of similarity (Euclidean, Pearson's correlation, Manhattan, etc.) and the resulting distances can be clustered with any of the usual methods (HC or K-means, for example).

There are some methods proposed in the recent literature that can handle arbitrary manifolds. In an early attempt to use manifold projection in clustering, Polito and Perona [41] showed how, in theory, LLE can naturally produce clusters. An interesting method (Path based clustering) based on graph theory was developed by Fisher and Buhmann in a series of papers [42-44]. They start by assuming that, if two points belong to the same cluster, there should be a path between them, going over other points in the same cluster, such that all distances in that path are small. They consider then that the length of a given path can be defined as the maximum distance between two points in the path. Following, they define the distance between two points as the minimum length among all the paths that connect the two points. We call this metric the Path Based Metric (PBM). The idea of measuring distances based on "neighbor of a neighbor" relations is the same we use. The main differences are that we use a k-nearest-neighbor graph, we consider all the edges in the path when we measure distances and we give a less extreme value to the maximum edge. PBM and the PKNNG metrics are basically heuristics, based on simple notions. There are also principled methods to find good metrics [45], learning the metric actively from the data and from examples of similar and dissimilar points provided by the final user. In principle the method can learn non-linear metrics, but it is not designed for data lying in manifolds. The well-known Single Linkage Hierarchical clustering [18] is efficient and has been widely used. This method is equivalent to finding the Minimum Spanning Tree (MST) of the dataset, and thus can also be considered as based on graphs.

Several more general but very efficient clustering methods have been introduced in the last years. We selected three of them to compare with our proposal of using a simple clustering method as PAM or HC with the new PKNNG metric. The Spectral Clustering algorithm [6,46] has recently received increasing attention and is considered to be effective for arbitrary manifolds using an appropriate kernel. The Evidence Accumulation Clustering algorithm (EAC) method of Fred and Jain [47] is based on the innovative idea of producing several different clustering solutions and extracting from them the information to produce the final clustering. The main idea is that if a pair of points is usually clustered together, under different conditions, then they could be assigned to the same cluster with high confidence. Recently, Kim et al. [48] have presented the Multi-K clustering methods, which is based on the same general idea of ensemble clustering.

In the next Section we show results using some artificial problems in high-dimensional spaces and discuss the application of the PKNNG metric to microarray datasets. Later, in the Methods Section, we explain in more detail the method and introduce and evaluate, in controlled experiments with other artificial datasets, the different schemes we use to connect the subgraphs and the diverse penalization functions considered in this work.

## Results and Discussion

### Evaluation on artificial datasets

In a first series of experiments we used artificial datasets to evaluate the behavior of the new metric in controlled situations, in which we change the difficulty of the clustering problem by setting, for example, the dimensionality of the input space or the distance between the clusters. In this and all other experiments in this work we consider that we know in advance the right number of clusters for each dataset (we discussed in the Introduction that we do not analyze the problem of finding the number of clusters in this work).

#### Experimental settings

We evaluated the proposed metric using five synthetic noisy datasets that simulate microarray samples:

##### A-1

The first dataset is taken from Kim et al. [48]. It is a 3 clusters problem in a 300 genes space, where each cluster has 50 samples. As explained by the authors, the 300 genes are divided into ten groups or blocks, each one with 30 genes. In each block for a given cluster all the 30 dimensional samples were commonly drawn from a Normal distribution *N*(*α*_30_, *I*_30_), where *α*_30 _is a 30-dimensional vector with all components equal to *α*, which is randomly chosen from {-0.5, 0, 0.5} in each block and *I*_30 _is the identity matrix in a 30 dimensional space. As explained by Kim et al., this dataset represents gene sets with co-expression patterns that are commonly up or down regulated under specific experimental conditions.

##### A-2

The previous dataset is a simple clustering problem, where all the clusters are well separated spheres. In this second artificial problem we start to consider the more complicated case of elongated clusters. This is a two clusters problem in a 100 genes space, where each cluster has 25 samples drawn from a Normal distribution. Each cluster has an elongated shape in the 100 dimensional space, where the principal direction (taken at random) explains 10% of the total standard deviation in the problem, and all the other dimensions have a similar deviation (the remaining 90% of the total standard deviation divided equally among the 99 directions). The principal axes of both clusters are parallel, separated by one and half times the deviation in the principal direction. This dataset simulates a problem in which all genes are highly correlated, but have a shift in their expression level for the two experimental conditions.

##### A-3

This is also a two clusters problem, generated as in A-2, where the centers of each cluster are in the same position as in A-2 (but separated by two times the deviation in the principal direction), but each of the two clusters has a different random direction for its principal axis. This dataset simulates a problem in which all genes are still correlated, but the correlation matrix is different for each experimental condition, which leads to a better separation when using correlation as base metric.

##### A-4

This is the three clusters version of problem A-3, where each of the three clusters has a different random direction for its principal axis. The separation among neighbor clusters is equal to the deviation in the principal direction.

##### A-5

This last dataset is similar to problem A-4, three elongated clusters with different correlation matrices and the same separation, but this time there are 200 genes that express differentially for the three conditions, and there are also 200 noisy genes (sampled from a centered Normal distribution with the same deviation used in all non-principal directions).

We considered two base metrics to evaluate similarities: Euclidean and Pearson's correlation. We evaluated distances with the PKNNG metric using 5 neighbors, MinSpan connection and exponential penalization. We first verified on reduced experiments with the artificial problems that the choice of the number of neighbors is not critical to the final clustering results. In the Methods Sections we discuss in particular the low dependence of the results with the connection and penalization methods, using other three artificial problems.

For comparison with the PKNNG metric we also used the PBM and RBF metrics. The PBM is a graph-based metric that we already described in the Introduction. It has no free parameters. The RBF or Gaussian metric is based on the RBF inner product 〈*x*, *z〉 *= *exp*(||*x *- *z*||)^2^/*σ*^2^), using the property of a kernel vector space that relates kernel to distance, ||*x *- *z*||^2 ^= 〈*x*, *x*〉 + 〈*z*, *z*〉 - 2〈*x*, *z*〉. This metric has a free parameter, *σ*, that acts as a global scale for the solution. We used two different procedures to set the value of *σ*. In the first case we set *σ *to the median of the base distance (Euclidean or correlation) among all pairs of points in the dataset. This follows the default strategy for model selection under the RBF kernel in the Kernlab R package [49]. We call this metric RBF-mean. As a second strategy, we used the procedure suggested by Ng et al [46] and implemented in the spectral clustering method in the same R package. In this case the criterion is to select the *σ *that gives the minimum within-cluster sum of square distances in the projected space for the spectral clustering method. We call this metric the RBF-min.

In all the experiments in this Subsection we use two of the most well-known clustering methods: Hierarchical clustering (HC) with average linkage and PAM, an appropriate version of K-means. For each evaluation we applied the following procedure: First we created a realization of the dataset. Then we measured distances in the dataset using the base, PBM, PKNNG, RBF-mean and RBF-min metric and applied HC and PAM to all distances. We evaluated the goodness of the solutions using the corrected Rand (cRand) index [50], also known as Adjusted Rand index (ARI), comparing the assigned cluster with the true label. For each dataset under study we produced 100 evaluations following this procedure and analyzed the corresponding distributions of cRand values.

#### Results

**Figure 1:**
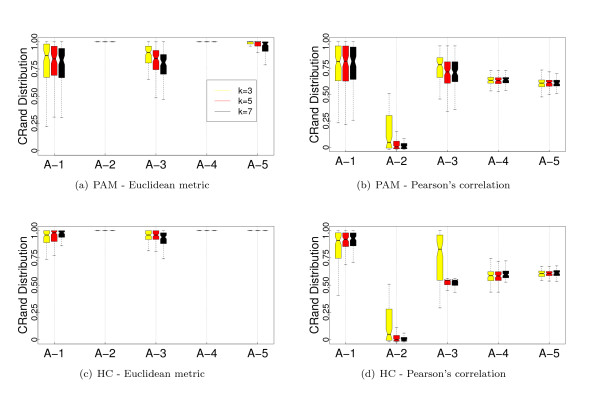
**PKNNG's dependence on *k*: Results on artificial datasets**. Evaluation of different number of neighbors (*k*) for the PKNNG metric. We use five artificial datasets that simulate gene expression problems and consider two clustering methods (PAM, top row, and HC, bottom row) and two base metrics (Euclidean, left column, and Pearson's correlation, right column).

**Figure 2:**
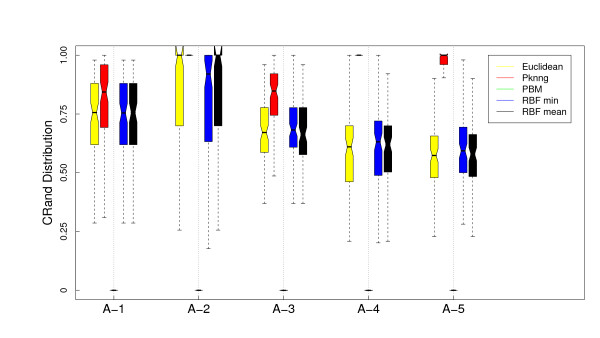
**Results on artificial datasets: Comparison with other graph-based metrics using PAM and the Euclidean base metric**. Evaluation of the PKNNG metric on five artificial datasets that simulate gene expression problems. For each dataset we show the results of the PAM clustering method using different metrics: plain Euclidean, PKNNG, PBM, RBF-mean and RBF-min.

**Figure 3:**
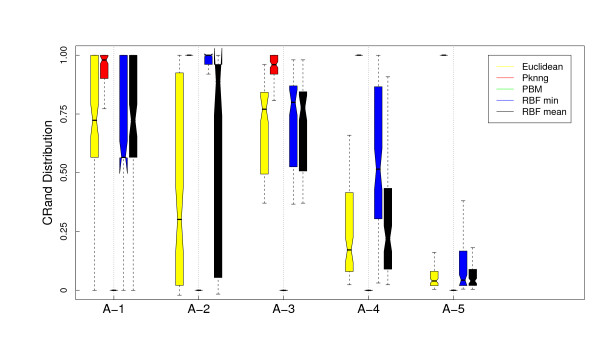
**Results on artificial datasets: Comparison with other graph-based metrics using HC (Av) and the Euclidean base metric**. Evaluation of the PKNNG metric on five artificial datasets that simulate gene expression problems. For each dataset we show the results of the HC clustering method (Average Linkage) using different metrics: plain Euclidean, PKNNG, PBM, RBF-mean and RBF-min.

**Figure 4:**
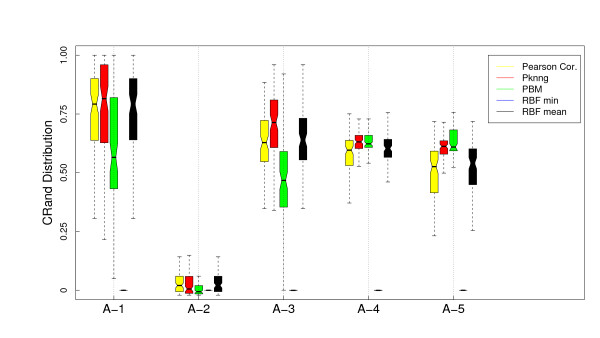
**Results on artificial datasets: Comparison with other graph-based metrics using PAM and the Correlation base metric**. Evaluation of the PKNNG metric on five artificial datasets that simulate gene expression problems. For each dataset we show the results of the PAM clustering method using different metrics: plain Pearson's correlation, PKNNG, PBM, RBF-mean and RBF-min.

**Figure 5:**
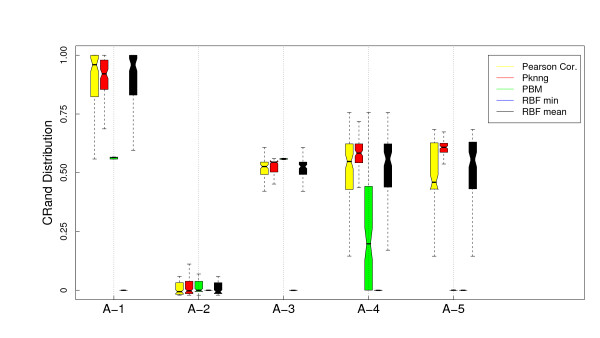
**Results on artificial datasets: Comparison with other graph-based metrics using HC (Av) and the Correlation base metric**. Evaluation of the PKNNG metric on five artificial datasets that simulate gene expression problems. For each dataset we show the results of the HC clustering method (Average Linkage) using different metrics: plain Pearson's correlation, PKNNG, PBM, RBF-mean and RBF-min.

Figure 1 shows the results of the analysis of the dependence of PKNNG with *k*, the number of neighbors considered in the graph, over the five artificial datasets using two different base metrics (Euclidean and Pearson's correlation) and two clustering methods (PAM and HC-average). For all the figures in this Section, the results are shown as boxplots of the distribution of cRand values corresponding to 100 experiments. For each distribution, the bottom and top of the box show the lower and upper quartiles, respectively, and the whiskers represent the lowest or highest datum still within 1.5 IQR of the corresponding quartile. Is is clear from the figure that the results are almost independent of *k*. In only 3 out of the 20 cases there is some dependence on *k*, all of them using the HC-average method. Figures 2, 3, 4 and 5 show the results of the comparison of the five metrics. In Figure 2 we use PAM clustering with distances based on the Euclidean metric. It is clear that in all cases the clustering results with the new PKNNG metric are clearly superior to those obtained with the plain Euclidean metric. Also, PBM shows a very poor performance, always similar to a random clustering. The PBM metric was designed to work in cases in which there are clear separations among the clusters, which is not the case in our artificial problems when using the euclidean base metric. When the clusters are not completely separated, PBM's base idea that only the longest edge counts for measuring distances in the graph leads to wrong results. PKNNG also usually gives a high weight to long edges in the graph, because in most cases they are the penalized ones, but it also considers the contribution of all other edges in the path connecting two points. In this sense, our metric can be viewed as a softened version of PBM. Both versions of the RBF metric show equivalent results, always similar to the base Euclidean metric in this case. Finally, all five datasets seem to be equally difficult using these settings.

In Figure 3 we show the results using the same base metric but with the HC clustering method. The results are very different this time, which highlights the importance of evaluating metrics with diverse clustering methods. For all metrics but the new PKNNG, HC tends to create one very small cluster in all problems (in fact, we had to force HC to discard clusters with less than 4 datapoints to obtain the non-random results in the figure). The PBM metric show the same poor results as with PAM. In the A-1 dataset the results are similar to PAM (Figure 2), but in the other four cases there is a big drop on performance for all metrics but the new PKNNG.

We also applied all methods using as base metric the Pearson's correlation. There are clear differences comparing with the Euclidean base metric. In Figure 4 we show the results for the PAM clustering method. RBF-min shows a very poor performance in this case, due to a bad automatic selection of the *σ *parameter. PBM, on the other side, is better suited for this setup, in which the clusters are more separated. In fact, PBM produces the best results for the A-5 dataset. Overall, PKNNG is still the best method but the differences are lower in this case. All methods show bad results for the A-2 dataset. This is simply due to the fact that, as the two clusters are colinear, there is no separation between them using Pearson's correlation. This is a clear limit for the class of metrics that we consider in this work, which are improvements of a given base metric and work by enhancing the separation among certain datapoints. The clusters in problems A-3, A-4 and A-5, which are elongated but no colinear, can be more easily separated by Pearson's correlation. The results with HC clustering, Figure 5 are qualitatively similar, but there are some differences in performance, in particular for the PBM metric in this case, which again shows the importance of evaluating metrics with diverse clustering methods.

Overall, considering the complete results on our artificial problems, PKNNG seems to be the more stable and efficient metric of the five compared in this work. It is interesting to note that the PKNNG metric produce accurate results both for elongated manifolds (A-2 to A-5) and for typical compact cloud data (A-1).

### Evaluation on gene expression datasets

In this second series of experiments we discuss the use of the PKNNG metric on real gene expression datasets.

#### Experimental settings

We included eight publicly available gene expression datasets in this analysis. Their main characteristics are summarized in Table 1. In the first seven datasets the objective is to cluster the different samples based on the information provided by the expression levels of a subset of genes. In these seven cases the samples have known classes corresponding to different tissues/phenotypes, measured by laboratory analysis (golden rule). In five of these datasets (ALB, LEU, BCLP, CNS and CGM) we used the reduced versions of Monti et al. [15]. In particular, for the CGM dataset we performed a second gene selection reducing the number of input genes to 1000, as in all the other datasets, considering the genes with the highest standard deviation. For the ALI dataset we used the version provided by De Souto et al. [12] and for the THY dataset we used the original version. In both cases we selected the subset of 1000 genes with the highest standard deviation. The last dataset (Y) is the only case in which the objective is to find four functional classes of genes based on information about different experimental conditions. In this case we used as input the original version of the dataset [20] and as golden rule the 4 functional classes selected by BenHur et al. [13] (one of the classes is in fact the union of two different functional classes). We included this dataset in our experimentation because it shows an intermediate gene clustering situation, with a fairly balanced examples/variables ratio (*n *~ *p*) and a few functional classes. Overall, we selected datasets with diverse difficulties, regarding the number of classes, the balance among them and the examples/variables (*n*/*p*) ratio. Following Monti et al. [15], we normalized all datasets by row to mean zero and standard deviation one (for each sample we normalize genes for the first seven datasets and experimental conditions for the last one).

**Table 1:** Gene Expression datasets

Dataset	n	Classes	p
AML-ALL [2] (ALB)	38	3 (11-8-19)	999
Alizadeh [5] (ALI)	62	3 (42-9-11)	1000
Leukemia [57] (LEU)	248	6 (43-27-15-79-20-64)	985
Novartis Tissue [58] (BCLP)	103	4 (26-26-28-23)	1000
CNS Tumors [59] (CNS)	48	5 (10-8-10-10-4)	1000
Normal Tissue [60] (CGM)	90	13 (5-9-7-11-6-7-6-5-12-10-4-5-3)	1000
Thyroid tumor GEO [GSE3467] (THY)	18	2 (9-9)	1000
Yeast [20] (Y)	208	4 (41-121-35-11)	79

Details on the eight gene expression datasets used in this work. We show the number of samples (n), variables (p) and the distribution of samples among the different classes.

We used the same experimental settings as with the artificial datasets. The only difference is in the sampling procedure. In this case, for each evaluation we took a sample with 95% of the examples in the dataset. Then we measured distances in the sample using all the metrics and applied the clustering method (HC or PAM) to all distances. For each dataset under evaluation we produced 100 evaluations following this procedure. Again, we evaluated the goodness of the clustering solutions using the cRand index, but this time comparing the assigned clusters with the original biological classes (golden rule). We also verified that there in no strong dependence on the value of *k*. We show the corresponding results in the additional file 1: Other evaluations.

We also applied three state-of-the-art clustering algorithms, described in the Introduction, to all eight gene expression datasets: i) The spectral clustering method with the RBF-min metric, ii) The EAC method with average linkage in its second step and iii) the recently introduced Multi-K clustering method. We also considered a fourth clustering method in this comparison, Model-Based Clustering (MBC) [51], which showed good results in gene-expression problems [52]. For this method we used a covariance structure consisting of a constant diagonal matrix for each cluster, but allowing a different constant for each cluster, which is the most complex structure that the method could use given the low number of samples in our gene expression datasets. In all cases we used the same protocol described in the previous paragraph for sampling and evaluation (in fact we used exactly the same 100 samples with 95% of the examples each one).

#### Results

**Figure 6:**
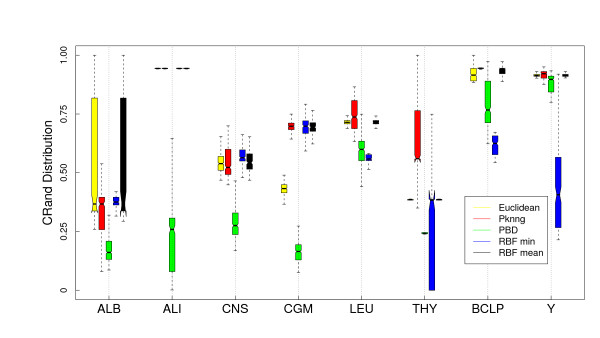
**Results on gene expression datasets: Comparison with other metrics using PAM and the Euclidean base metric**. Evaluation of the PKNNG metric on eight gene expression datasets. For each dataset we show the results of the PAM clustering method using different metrics: plain Euclidean, PKNNG, PBM, RBF-mean and RBF-min.

**Figure 7:**
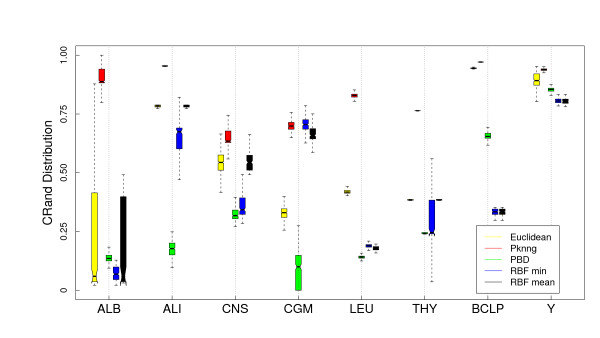
**Results on gene expression datasets: Comparison with other metrics using HC (Av) and the Euclidean base metric**. Evaluation of the PKNNG metric on eight gene expression datasets. For each dataset we show the results of the HC clustering method (Average Linkage) using different metrics: plain Euclidean, PKNNG, PBM, RBF-mean and RBF-min.

**Figure 8:**
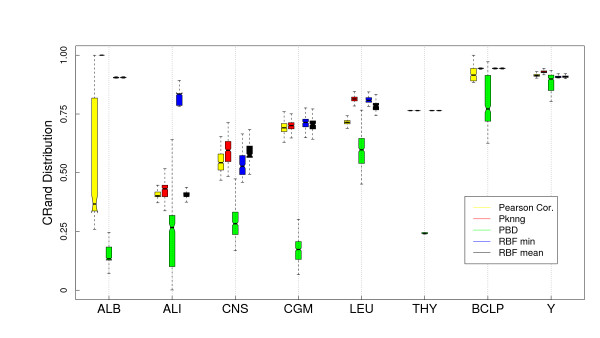
**Results on gene expression datasets: Comparison with other metrics using PAM and the Correlation base metric**. Evaluation of the PKNNG metric on eight gene expression datasets. For each dataset we show the results of the PAM clustering method using different metrics: plain Correlation, PKNNG, PBM, RBF-mean and RBF-min.

**Figure 9:**
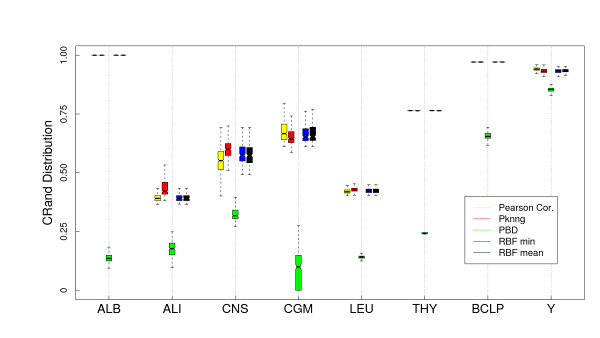
**Results on gene expression datasets: Comparison with other metrics using HC (Av) and the Correlation base metric**. Evaluation of the PKNNG metric on eight gene expression datasets. For each dataset we show the results of the HC clustering method (Average Linkage) using different metrics: plain Correlation, PKNNG, PBM, RBF-mean and RBF-min.

In Figure 6 we show a comparison of the five metrics using the PAM clustering algorithm and the Euclidean base metric on the eight gene expression datasets considered in this work. PKNNG shows the more consistent behavior. In seven out of the eight datasets it is equivalent or superior to the other metrics. PBM and RBF results are much more problem-dependent. In Figure 7 we show the same experiment but using HC as clustering method. The differences in favor of PKNNG are clearer in this case. The combination of HC and the PKNNG metric shows the biggest improvements in accuracy over HC-Plain, in particular for the ALB, ALI, LEU and THY datasets. Smaller improvements can be observed for the CNS, BCLP and Y datasets. On the two figures it is notorious that in several cases PBM or RBF show results that are lower than the base metric. In Figures 8 and 9 we show the corresponding experiments using Pearson's correlation as base metric. Apart from the PBM metric, which always shows a bad performance, the remaining four metrics show a more stable result with this base metric. PKNNG show only small improvements over the plain metric in this case. When using the PAM method, PKNNG is the only metric that finds the correct solution for the ALB dataset. It also shows interesting results for the LEU and Y datasets. The ALI dataset with these settings is the only case in all our experiments in which another metric (RBF-min) clearly outperforms PKNNG.

**Figure 10:**
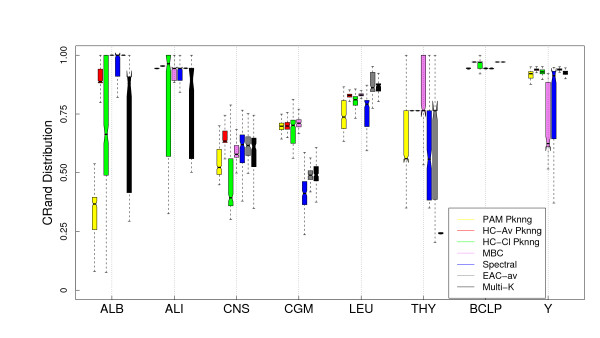
**Results on gene expression datasets: Comparison with other state-of-the-art clustering methods (Euclidean base metric)**. Evaluation of the PKNNG metric on eight gene expression datasets using the base Euclidean metric. For each dataset we compare our proposal (the PKNNG metric plus PAM or HC) with other four methods: Model-Based Clustering (MBC), Spectral clustering (Spectral), Evidence accumulation with average linkage (EAC-av) and the Multi-K method.

**Figure 11:**
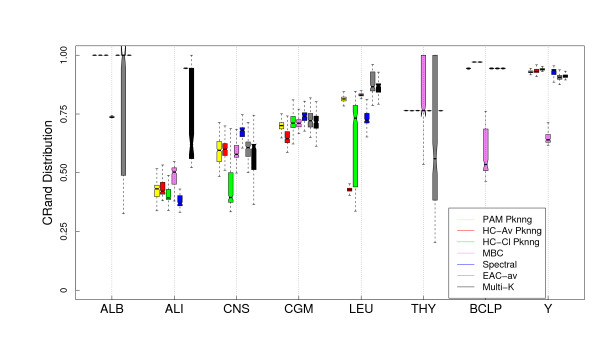
**Results on gene expression datasets: Comparison with other state-of-the-art clustering methods (Correlation base metric) Evaluation of the PKNNG metric on eight gene expression datasets using Pearson's correlation as base metric**. For each dataset we compare our proposal (the PKNNG metric plus PAM or HC) with other four methods: Model-Based Clustering (MBC), Spectral clustering (Spectral), Evidence accumulation with average linkage (EAC-av) and the Multi-K method.

In Figures 10 and 11 we show the comparison of our proposal (to use the PKNNG metric plus a simple clustering method like HC or PAM) with four state-of-the-art clustering algorithms for the Euclidean and Pearson's correlation base metrics. In this case we also included other version of HC, the complete linkage method. For the Euclidean base metric, Figure 10 in six out of the eight datasets one of our proposals shows the best results (or equivalent to the best method) in extracting the biological information related to the original classes/tissues. Only for the ALB and LEU datasets some other method clearly outperforms our proposal. The same analysis is valid when using correlation as base metric (Figure 11). The EAC and Multi-K methods outperforms all other methods in the ALI and LEU datasets and Spectral clustering shows a good result in the CNS dataset. Making a global evaluation, considering all datasets and base metrics, there are no clear winners or losers, all the methods are competitive in some situations. As we stated before, we are comparing efficient methods in these experiments and, in consequence, which one works better is highly dependent on the dataset and the base metric.

For completeness, in the Additional file 2: Evaluation with different number of clusters we show the results of changing the number of selected clusters in the gene expression datasets with all metrics and clustering methods. Those figures show that in almost all cases cRand results (for all the metrics and methods discussed in this work) have a simple dependence with the number of cluster, showing a peak at the right place for each problem and a smooth decay after that point. Of course, the results in those figures cannot be used to select the right number of clusters for each dataset, as the cRand measures are based in a previous knowledge of the golden rules.

## Conclusions

In this work we have discussed the Penalized k-Nearest-Neighbor-Graph based metric and have shown that it is a useful tool for clustering arbitrary manifolds. The PKNNG metric is based on a two-step method: first it constructs the k-Nearest-Neighbor-Graph of the dataset using a low k-value (from 3 to 7), and then it uses penalized weights for connecting the sub-graphs produced by the first step. In the Methods section we clearly show that the key factor in the good performance of the PKNNG metric is the use of highly penalized weights at the second step.

We evaluated our proposal using five artificial datasets with different difficulties (that simulate gene expression problems) and eight public gene expression datasets. We showed first that our new metric is superior to other graph-based metric previously introduced and to the base metrics (Euclidean and Pearson's correlation) in all cases under analysis. In second term we showed that our proposal produced an equivalent performance to other state-of-the-art clustering methods on all the wide gene expression datasets evaluated in this work.

The use of the PKNNG metric can improve the performance of commonly used pairwise-distance based clustering methods, to the level of more advanced algorithms. A great advantage of the new procedure is that researchers do not need to learn a new method, they can simply compute distances with the PKNNG metric and then, for example, use hierarchical clustering to produce an accurate and highly interpretable dendrogram of their high-dimensional data.

## Methods: The PKNNG metric

The evaluation of similarities with the PKNNG metric is a two-step process. First we search the original dataset space for locally dense (connected) structures using the knn-graph. In the ideal case the process should end with a connected subgraph corresponding to each cluster but in the real case, when working with finite noisy samples, there are usually too many separated structures, typically more than one for each real cluster. In the second step we add penalized edges to the graph in order to fully connect it, and use an appropriate algorithm to measure distances in the (now) connected graph.

### First step: knn-graphs

**Figure 12:**
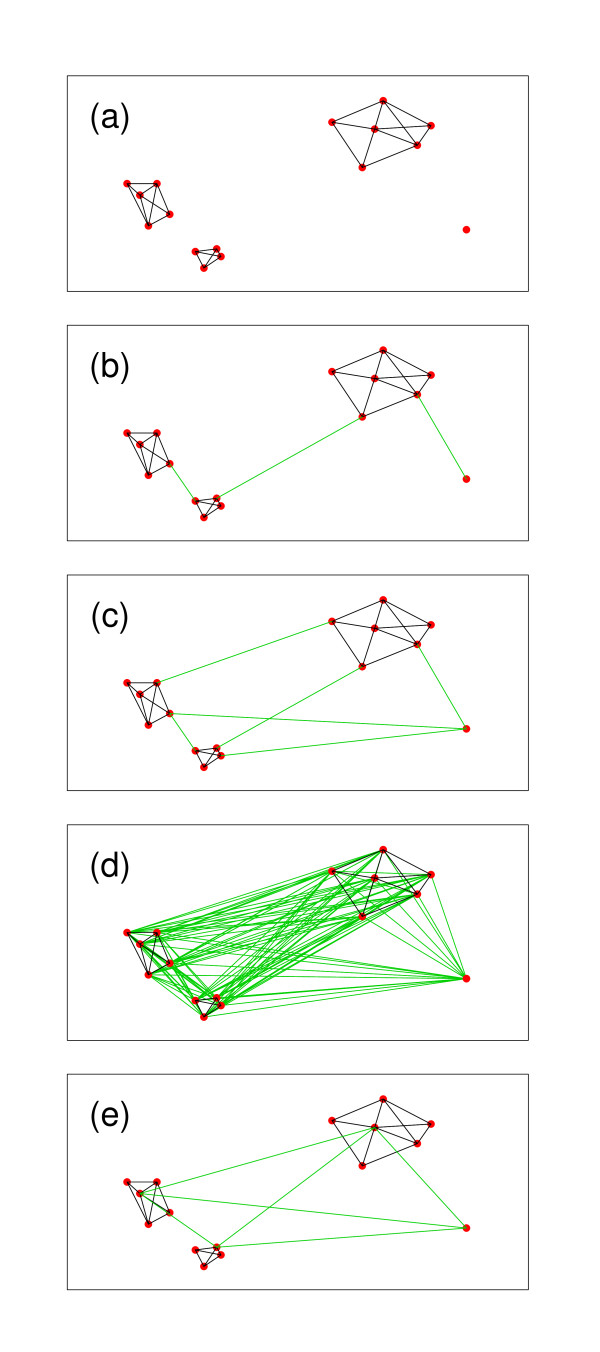
**Connection schemes**. A toy dataset illustrating the different connection schemes evaluated in this work. a) The original data with the knn-graph. Note the disconnected outlier at the bottom-right. b) The MinSpan scheme. c) The AllSubGraphs scheme. Note that all MinSpan added edges are included. d) The AllEdges scheme. e) The Medoids scheme. See text for details on each method.

Among the several algorithms for discovering low dimensional manifolds recently introduced, ISOMAP has strong theoretical properties and is also easy to understand. We follow the main ISOMAP idea to search for locally connected structures. As explained by Tenenbaum, de Silva and Langford [38], in a curved manifold the geodesic distance between neighboring points can be approximated by the Euclidean input space distance. For distant points, in contrast, geodesic distances are better approximated as a path of short segments connecting neighboring points. To this end, we construct the knn-graph of the data, i.e. the graph with one vertex per observed example, arcs between each vertex and its *k *nearest neighbors, and with weights equal to the Euclidean distance between them. As we look for dense subgraphs, at the end of the process we eliminate all the outliers from the graphs. We consider that an arc is an outlier if it is not reciprocal (i.e. one of the vertex is not a k-nn of the other) and the length of the arc is an outlier of its own distribution (i.e. if its length is larger than the 3rd quartile plus 1.5 times the inter-quartile distance of the distribution of the lengths of all the edges in the graph). As isolated points usually produce not reciprocal connections in k-connected-graphs, with this definition we are basically deleting long connections from these kind of points. In Figure 12 we show a toy example of this process. Panel (a) shows the original data with the corresponding knn-graph. The isolated point at the bottom left corner is an outlier, and the edge connecting it with the nearest sub-graph has been eliminated. In general, the use of a low number of neighbors (3 to 7 in all our cases, as in the original work [38]) produces graphs that can follow the curved structure of any data without adding "shortcuts" between geodesically distant points (a known issue with ISOMAP [53]), but usually paying the price of producing disconnected components [53], which we connect in the next step.

### Second step: connecting subgraphs

As stated previously, when working with real data the first step will usually generate separated structures, typically more than one for each real cluster. In the ideal case, each sub raph should correspond to a unique cluster and we could leave them disconnected and interpret those distances as equal to infinite, as done for example by Brito et al. [54]. In the real case, however, some sub-graphs correspond to the same cluster or two cluster could lie in the same subgraph. Therefore, we need to evaluate also geodesic distances between points lying in different sub-graphs. With that purpose, we add to the graph a number of edges in order to fully connect it.

#### Connection schemes

Which edges to add in order to connect the graph is an interesting problem by itself. We evaluated in this work four different schemes that cover most of the simple possibilities:

##### MinSpan

In this first scheme we add to the graph the minimum number of edges, each of them of minimum length (the minimum spanning set), which fully connect the graph. On panel (b) of Figure 12 we show the result of this connection strategy on our toy example.

##### AllSubGraphs

Here we connect each sub-graph to all other sub-graphs using minimum length edges. Figure 12 panel (c), shows this strategy on the toy example.

##### AllEdges

In this simple scheme we add to the graph all remaining edges (of course, with a penalized weight). On panel (d) of Figure 12 we show the corresponding graph.

##### Medoids

In this last strategy we first find the medoid of each sub-graph, and then add edges connecting each medoid to all remaining medoids. Figure 12 panel (e), shows this strategy applied to our toy example.

The idea behind MinSpan is to add the shortest available edges trying to follow as much as possible the structure of the manifold. MinSpan basically produces one-dimensional structures. We showed in a previous work [55] that this effect can introduce some instability for distant points, but it does not affect the performance of clustering. AllSubGraphs is an extension of MinSpan and AllEdges can be viewed as an extension of AllSubGraphs. At each step of this chain we add more edges, which increases the connectivity of the graph, reducing the potential instability of MinSpan. On the other hand, by adding edges we increase the probability of introducing "shortcuts" in the manifold, which can reduce the overall performance of the method. The Medoids scheme is basically introduced to have a different scheme to compare with. As it does not include the minimum spanning set, it is always forced to use other paths in the graph and, consequently, it is not expected to be able to follow a curved manifold. Neighboring points belonging to different sub-graphs can be completely separated by this connection scheme.

Once we have a connected graph, we can compute geodesic distances between all points as minimum-length paths in the graph using computationally efficient algorithms like Floyd or Dijkstra [56]. This is the most time consuming stage in the computation of the metric, with a complexity of *O*(*n*^2^*logn*), where *n *is the number of points in the dataset. It is interesting to note that the number of dimensions in the problem only enters in the initial computation of the distance among all points, which is a mandatory step for any clustering method. The burden that we are adding is only related to the number of points in the datasets, and is lower than the complexity of most hierarchical methods. Furthermore, in this work we analyze gene expression datasets, for which *n *is usually a low number.

In order to compare these four connection schemes in controlled situations we used three artificial datasets with different characteristics:

##### Two-moons

The first dataset corresponds to points uniformly sampled from two arcs of circumference (two clusters), with Gaussian noise of a fixed amplitude added to the radial direction.

##### Three-spirals

The second artificial dataset was generated by sampling uniformly from three equally separated spirals, with added Gaussian noise proportional to the radial distance. The result is a three clusters problem, each one of them having a non-uniform density, which can confuse some algorithms.

##### Three-rings

On this problem the central cluster corresponds to a uniform sampling of a circle, which is surrounded by two rings, also sampled uniformly but with constant Gaussian noise added to the radial component. We split both middle- and outer-rings in halves (adding a small gap), to create a more difficult five clusters problem. This third dataset has clusters with different (but uniform) densities.

**Figure 13:**
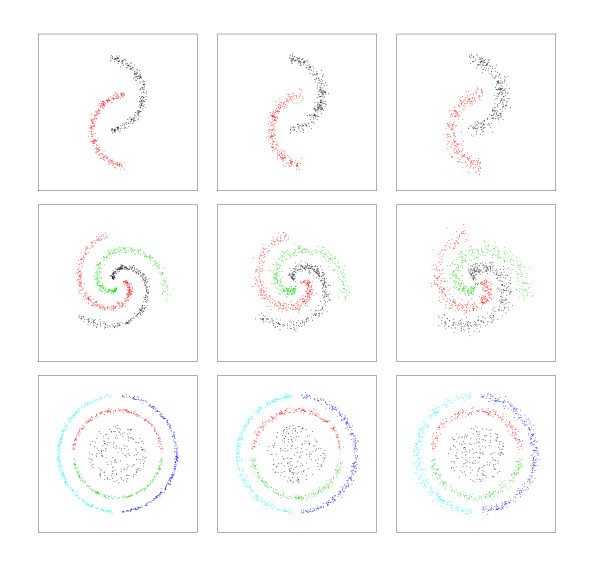
**Artificial datasets**. Samples of the artificial datasets used in this work. From top to bottom, rows correspond to the Two-moons (2 clusters), Three-spirals (3 clusters) and Three-rings (5 clusters) datasets. From left to right, columns correspond to low, medium and high noise levels, respectively.

Similar datasets were used by other author with the same goal [46-48]. All datasets were generated in a (2-dimensional) plane. For each one of them we used three different noise levels. An example of the artificial clustering problems is shown in Figure 13. In order to evaluate the performance of the PKNNG metric at handling low-dimensional manifolds in high-dimensional spaces, we embedded our three datasets in 2D, 3D and 10D spaces, generating the following four settings:

##### 2D

In this first case we kept the three datasets in the original 2D space.

##### 3D

**Figure 14:**
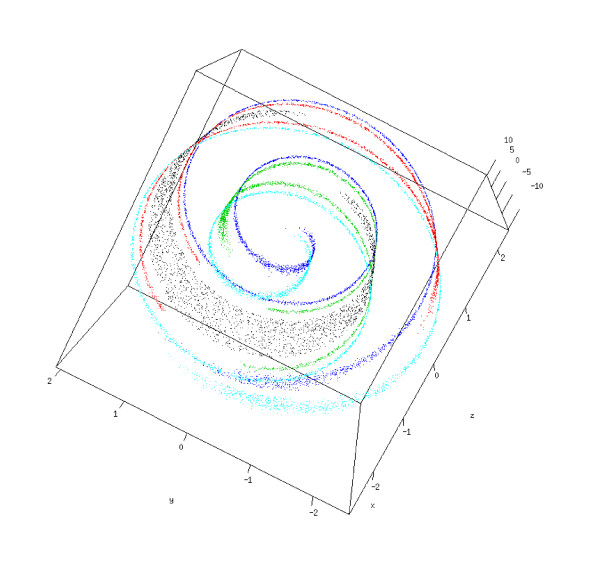
**Swiss-roll embedding**. Example of the embedding of our 2D artificial clustering problems in a 3D swiss-roll.

To start increasing the difficulty of the clustering problems, we coiled the original plane to form a swiss-roll, producing a non-linear embedding of the original clusters into a 3D space. In Figure 14 we show an example of the resulting problem.

##### 3D-noise

In this third setting we added Gaussian noise to the previous 3D embedding in order to drift the points away from the surface of the swiss-roll.

##### 10d-noise

As a last and more difficult setting, we took the 3D coiled data and added 7 extra dimensions to the problem. We then applied a random rotation in the 10D space, and finally added Gaussian noise in all 10 dimensions.

In all cases we measured base similarities with the Euclidean metric. For these artificial datasets the quality of the solutions was evaluated in terms of the clustering accuracy, i.e. the percentage of the dataset assigned to the right cluster. For each case under evaluation (dataset + noise level + embedding) we produced 100 different realizations of the dataset and computed the mean clustering accuracy.

**Figure 15:**
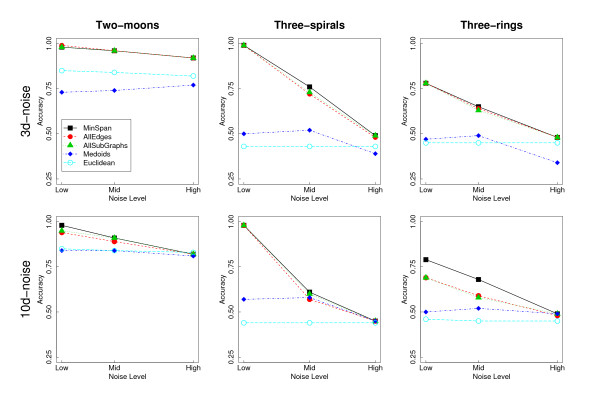
**Comparison of the different connection schemes**. Comparison of the four different connection schemes evaluated in this work: Min-Span, AllSubGraphs, AllEdges and Medoids. Columns of sub-figures correspond, from left to right, to the Two-arcs, Three-spirals and Three-rings datasets. Rows correspond to the two more difficult embeddings; from top to bottom: 3d-noise and 10d-noise. Each sub-figure shows the mean clustering accuracy of the diverse methods at three different noise levels.

We used only two of these embeddings (3D-noise and 10d) to compare the four connection schemes here (we show the results of the four embeddings in the additional file 3: Full Figures). In Figure 15 we show the corresponding results using the PAM clustering algorithm and the exponential penalization (discussed in the next Subsection). We repeated the full experiment using the HC clustering algorithm, finding completely equivalent results (data not shown). At each panel we also include the results of using the standard Euclidean metric as a baseline reference.

The qualitative results are similar in all the situations under analysis. It is clear from the figure that three connection schemes (MinSpan, AllSubGraphs and AllEdges) have very similar performances, clearly superior to the baseline methodology. The Medoids scheme, as expected, does not show a real improvement over the plain Euclidean metric. Comparing the three efficient schemes, MinSpan seems to be slightly superior to AllSubGraphs and AllEdges. Based in these results we selected the Minspan connection scheme in the general evaluation with real and artificial datasets (Results and Discussion Section).

##### Weighting schemes

The key point of the PKNNG metric is that we penalize the weight of these new connecting edges. The logic behind penalizing is simple: the edges added by the different connection schemes lie on very low density sections of the space and going through those regions when measuring distances is opposite to the basic idea that the graph connects high density regions. Then, we penalize the added edges in order to clearly differentiate paths that need to go through low density regions from those who only use high density regions.

We found that a very effective penalization is the use of an exponential factor of the form:

(1) $$w=d\;\;e^{d/\mu },$$

where *w *is the graph weight corresponding to the added edge, *d *is the Euclidean distance between the points being connected by that edge and *μ *is the mean edge weight in the original graph. Using this metric we can connect sub-graphs corresponding to the same cluster with a relatively small cost, because connections in the same order of magnitude of *μ *will get a low penalization. On the other hand, edges connecting distant sub-graphs will be strongly penalized. In the additional file 4: Evaluation of Penalization Functions we show results of experiments with several penalization functions, including linear (*w *= *αd*, with *α *∈ ℜ^+^), power (*w *= *d *(*d*/*μ*)*^k^*, with *k *∈ *N *^+^) and other exponential functions, and different definitions of *μ*. Those results show that the functional form of the penalization is not critical to the method, given that it highly penalizes long edges.

**Figure 16:**
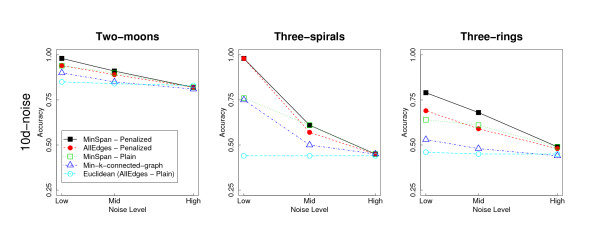
**Penalization**. Comparison of two connection schemes (MinSpan and AllEdges) in their "plain" and exponentially penalized forms, using the 10D embedding. Columns of sub-figures are the same as in Figure 15.

Figure 16 shows an experiment performed to assess the real influence of the penalization on the PKNNG metric. In this case we included in the comparison two connection schemes, namely MinSpan and AllEdges, under two different settings: i) in the (normal) exponentially penalized version (as used in all previous experiments), and ii) in a "plain" version in which we eliminated the penalization and used for the added connecting edges the standard Euclidean distance. Both settings used exactly the same graphs, the only difference being the high penalization of some weights in the first case. It is easy to see that the "plain" version of AllEdges is simply the standard Euclidean metric, our baseline in all experiments. For completeness, we also include in this case the results obtained measuring similarities with the knn-graph with the minimum k-value that produces a fully connected graph (we call this method min-k-connected-graph). In Figure 16 we show the results obtained with the PAM clustering algorithm applied to the five settings described in this paragraph for the 10D embedding (in the additional file 3: Full Figures we show the complete figure with the four different embeddings). Analyzing the figure, the two methods that use "plain", non-fully-connected Euclidean graphs (the MinSpan-Plain and the min-k-connected-graph) show in most cases a better performance than the standard Euclidean metric, but, more importantly, the two penalized metrics are in all cases clearly superior to the corresponding "plain" methods. These results suggest that the key factor of our new metric is the high penalization of the added edges.

Finally, at this point we can consider the problem of outlier datapoints. After the First Step, our method left all outlier datapoints disconnected to any subgraph. If we apply the Second Step as it was described in the last paragraphs, we will end with penalized connections to outlier datapoints. In this case, most clustering algorithms will create individual clusters for each outlier. The advantage of this solution is that outliers are clearly identified, but, on the other hand, the number of clusters is artificially increased. Most researchers prefer another treatment for outliers, to add them to the nearest cluster. This can be easily done with our metric, by taking into account the number of datapoints in a subgraph before penalizing a connection. If the edge connects to a single datapoint, we just add an edge with no penalization. As clustering method usually separate clusters by the penalized edges, in most cases outliers will be assigned to the nearest cluster. We used this setting in all the experiments in this work.

## Availability

An R implementation of the PKNNG metric can be download from http://www.cifasis-conicet.gov.ar/granitto/nng_0.0.1.tgz or requested to the contact author. All dataset in this work can also be requested to the contact author.

## Authors' contributions

AB implemented all methods and performed all statistical analysis, PMG wrote the manuscript, both authors devised and developed the method and read and approved the manuscript.

## Supplementary Material

Additional file 1 **Other evaluations**. Evaluation of: i) dependence on *k *for public gene expression datasets and ii) the use of HC-complete linkage with our five metrics. Click here for file

Additional file 2 **Evaluation with different number of clusters**. Evaluation of the effect of changing the number of selected clusters in the gene expression datasets with all metrics and clustering methods. Click here for file

Additional file 3 **Full Figures**. Complete versions of Figure 15 and 16, including the four embeddings discussed in the Methods section. Click here for file

Additional file 4 **Evaluation of Penalization Functions**. Discussion and evaluation of other penalization functions. Click here for file
