# Supplementary material for: Clustering gene expression data with a penalized graph-based metric
Source: BMC Bioinformatics. 2011 Jan 4;12:2. doi: 10.1186/1471-2105-12-2 (PMC3023695; doi:10.1186/1471-2105-12-2)
Supplement: Additional file 1 — Other evaluations. Evaluation of: i) dependence on k for public gene expression datasets and ii) the use of HC-complete linkage with our five metrics. [file 1471-2105-12-2-S1.PDF]

Clustering gene expression data with a penalized graph-based metric

A. Baya & P.M. Granitto

Other evaluations

This file includes a series of figures evaluating: i) the dependence on k for public gene expression datasets, ii)the use of HC-complete linkage with our five metrics. In all cases the experimental setups and the evaluations are similar to those described in the paper.

i) Dependence on k for public gene expression datasets.

In Figures 1 and 2 we show the dependence on k, the number of neighbors in the graph, for the PKNNG metric using the public datasets. For the Euclidean base metric, Figure 1, the results are completely stable in five out of the eight datasets. For the ALB, ALI and THY datasets the results of the metric change with k. This three particular problems are very difficult and unstable for all methods, not only for PKNNG, as can be observed in Figures 6 and 10 in the paper. The results for Pearson's correlation, Figure 2, are stable in almost all cases.

ii) Results with HC-complete linkage

In Figure 3 we show the comparison of the five metrics analyzed in the paper on the public gene expression datasets. The results are qualitatively similar to other clustering methods.

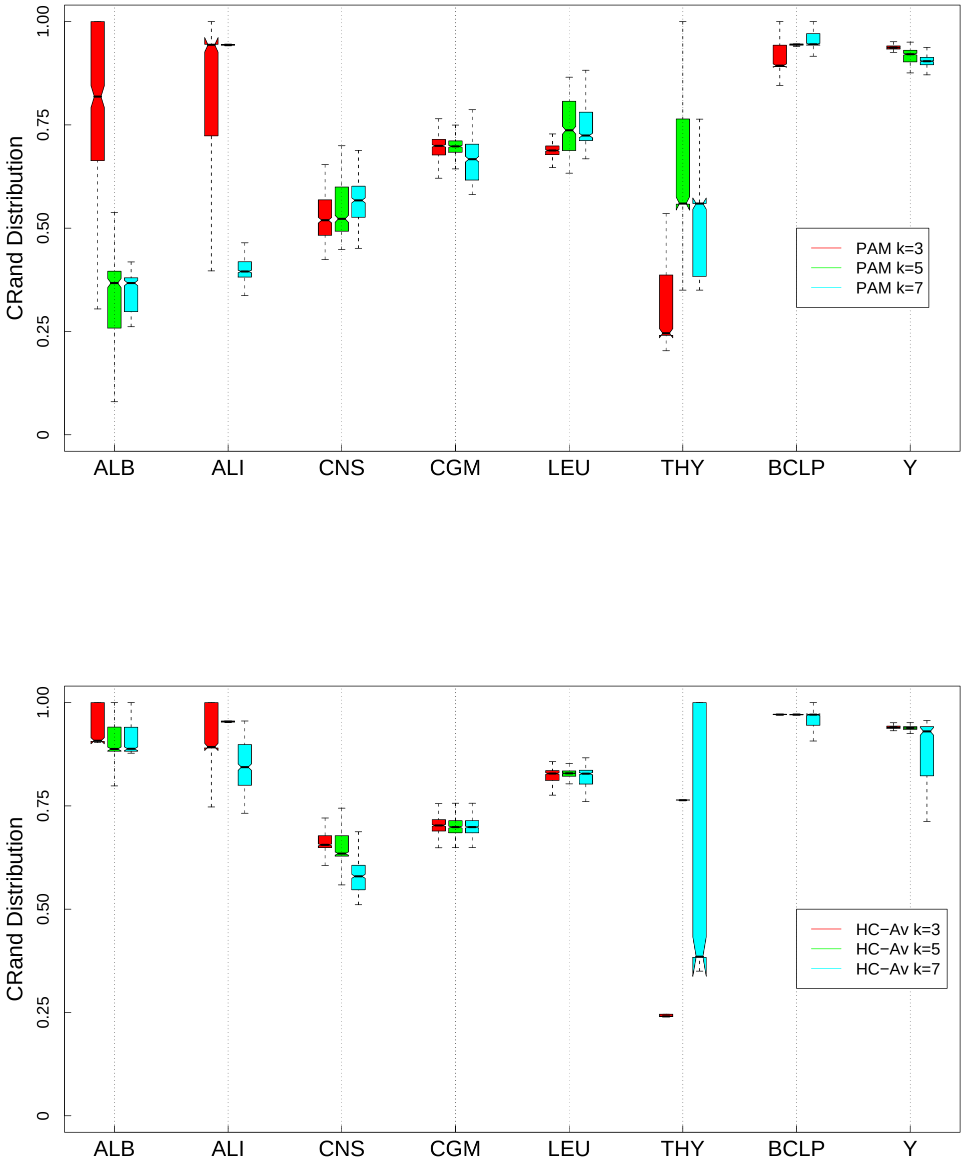

Figure 1: Evaluation of different number of neighbors (k) for the PKNNG metric. We use eight public gene expression datasets and consider two clustering methods (PAM, top, and HC, bottom) for the Euclidean base metric. The results are distribution of cRand values over 100 experiments, as in Figures 6 to 9 in the paper.

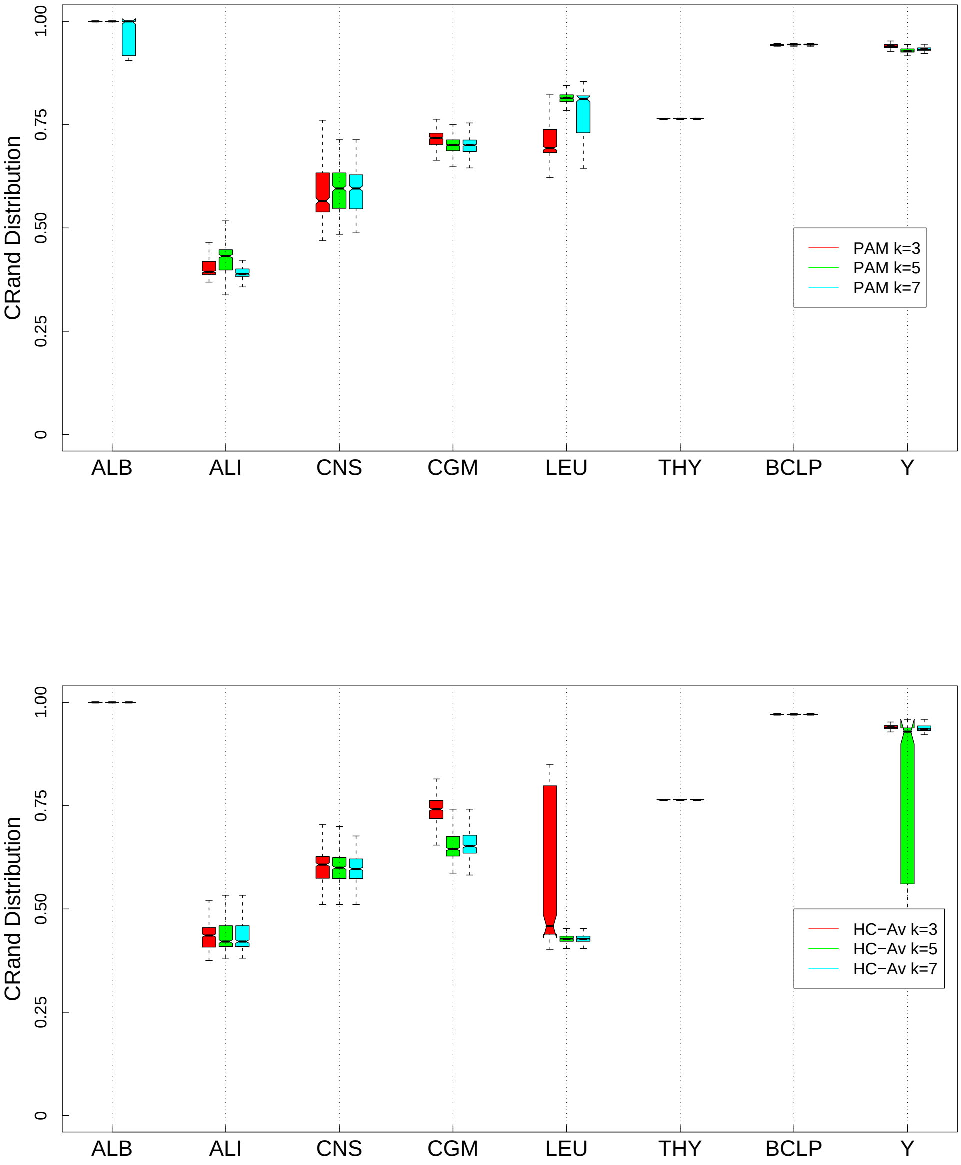

Figure 2: Evaluation of different number of neighbors (k) for the PKNNG metric. We use eight public gene expression datasets and consider two clustering methods (PAM, top, and HC, bottom) for the Pearson's correlation base metric. The results are distribution of cRand values over 100 experiments, as in Figures 6 to 9 in the paper.

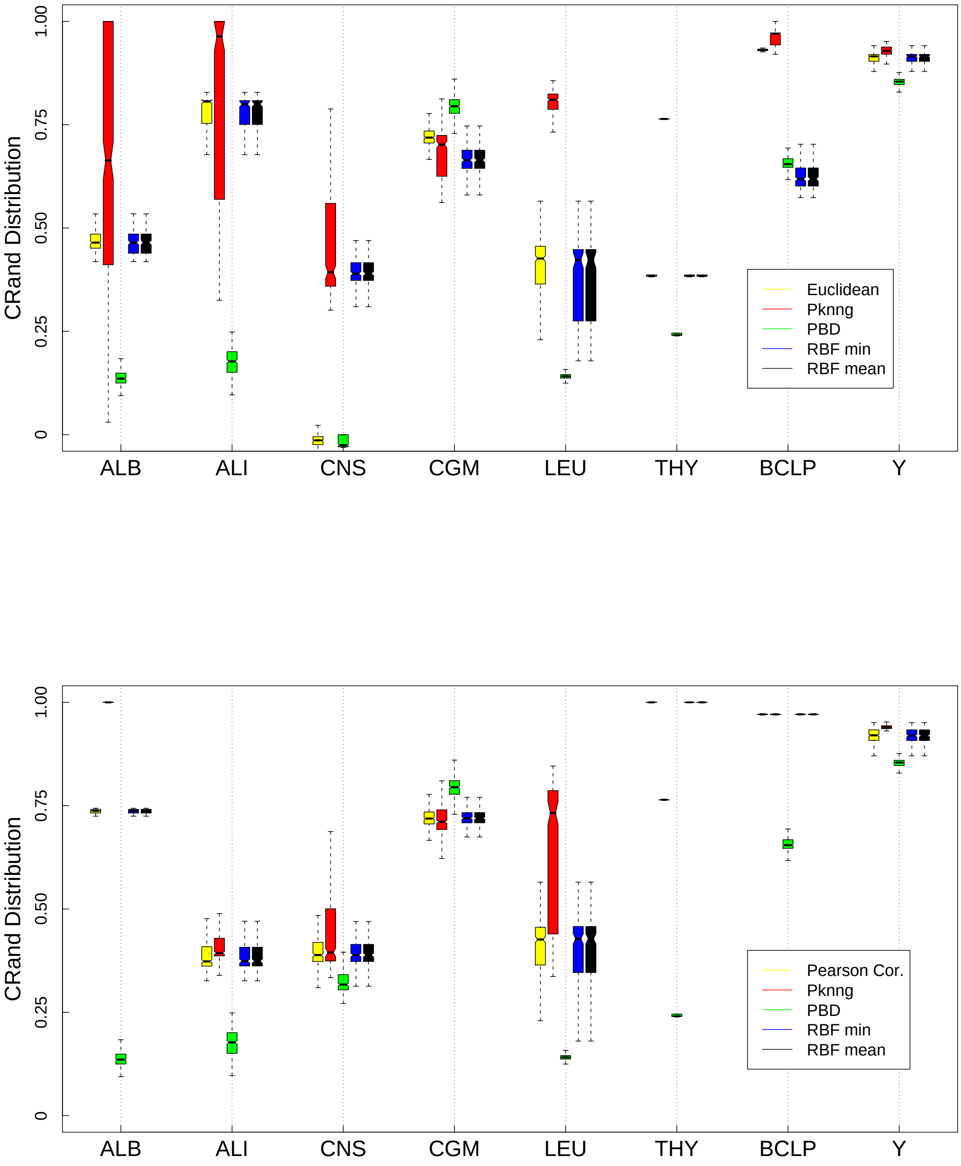

Figure 3: Results on gene expression datasets: Comparison with other graph-based metrics using HC (Complete linkage). Evaluation of the PKNNG metric on eight public gene expression datasets. For each dataset we show the results of the HC clustering method (Complete Linkage) using different metrics: plain Euclidean, PKNNG, PBM, RBF-mean and RBF-min.
