# Supplementary material for: Clustering gene expression data with a penalized graph-based metric
Source: BMC Bioinformatics. 2011 Jan 4;12:2. doi: 10.1186/1471-2105-12-2 (PMC3023695; doi:10.1186/1471-2105-12-2)
Supplement: Additional file 2 — Evaluation with different number of clusters. Evaluation of the effect of changing the number of selected clusters in the gene expression datasets with all metrics and clustering methods. [file 1471-2105-12-2-S2.PDF]

Clustering gene expression data with a penalized graph-based metric

A. Baya & P.M. Granitto

Evaluation with different number of clusters.

This file includes a series of figures evaluating the effect of changing the number of selected clusters. The reader should take into account that we are using cRand as quality measure, comparing each solution with the golden rule (fixed number of classes) in each case. In consequence, these figures are not equivalent to those typically used to evaluate gene clustering against external information, as for example gene ontology. Also, they cannot be used to search for the right number of clusters in a problem, as they use information about the original classes in the problem.

In all cases the experimental setups and the evaluations are similar to those described in the paper.

In the first series of figures we show the results of changing the number of clusters selected by PAM or HC on the eight gene expression datasets. First we show results for the five metrics evaluated in the main text: The base metric, PKNNG, PBM and the two versions of the RBF metric. In all figures we show the mean cRand value over 100 experiments (as described in the paper) as a function of the number of clusters extracted. In Figures 1 to 8 we show results using the Euclidean base metric. Then, in Figures 9 to 16 we show the corresponding results for the Pearson's correlation base metric.

When using the HC clustering method, there are situations in which the method cannot find a solution with the required number of cluster (understanding that a singleton is not a cluster). In that cases we report a cRand value of zero.

In a second series of figures we show the results of using several clustering methods on the same 8 real world gene-expression datasets. Again, in all figures we show the mean cRand value over 100 experiments (as described in the paper) as a function of the number of clusters extracted. In Figures 17 to 24 we show results using the Euclidean base metric. Last, in Figures 25 to 32 we show the corresponding results for the Pearson's correlation base metric.

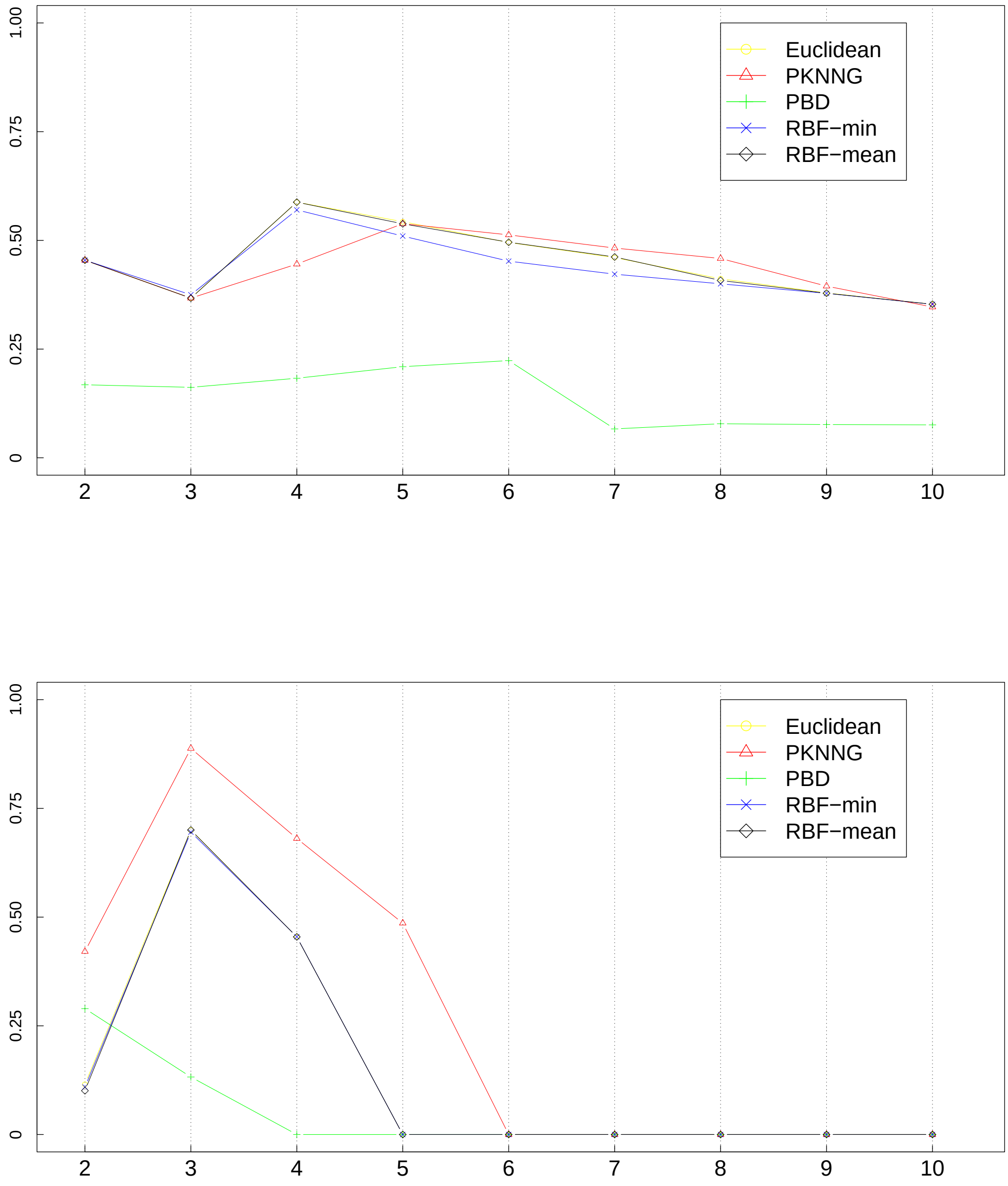

Figure 1: Evaluation of different metrics as a function of the number of clusters extracted for the ALB gene expression dataset (3 classes) using the Euclidean base metric. Top panel: PAM clustering. Bottom panel: HC-av clustering.

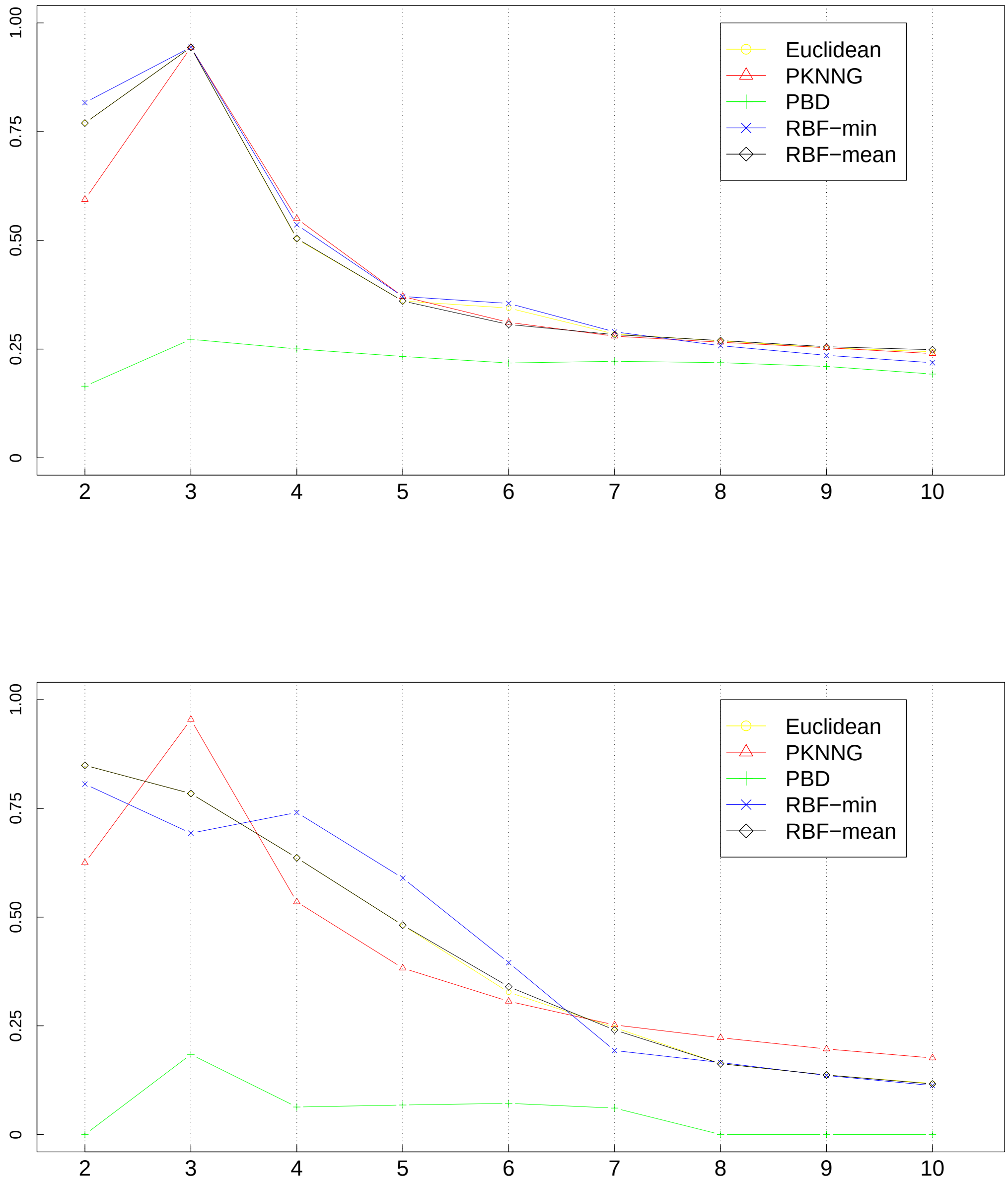

Figure 2: Evaluation of different metrics as a function of the number of clusters extracted for the ALI gene expression dataset (3 classes) using the Euclidean base metric. Top panel: PAM clustering. Bottom panel: HC-av clustering.

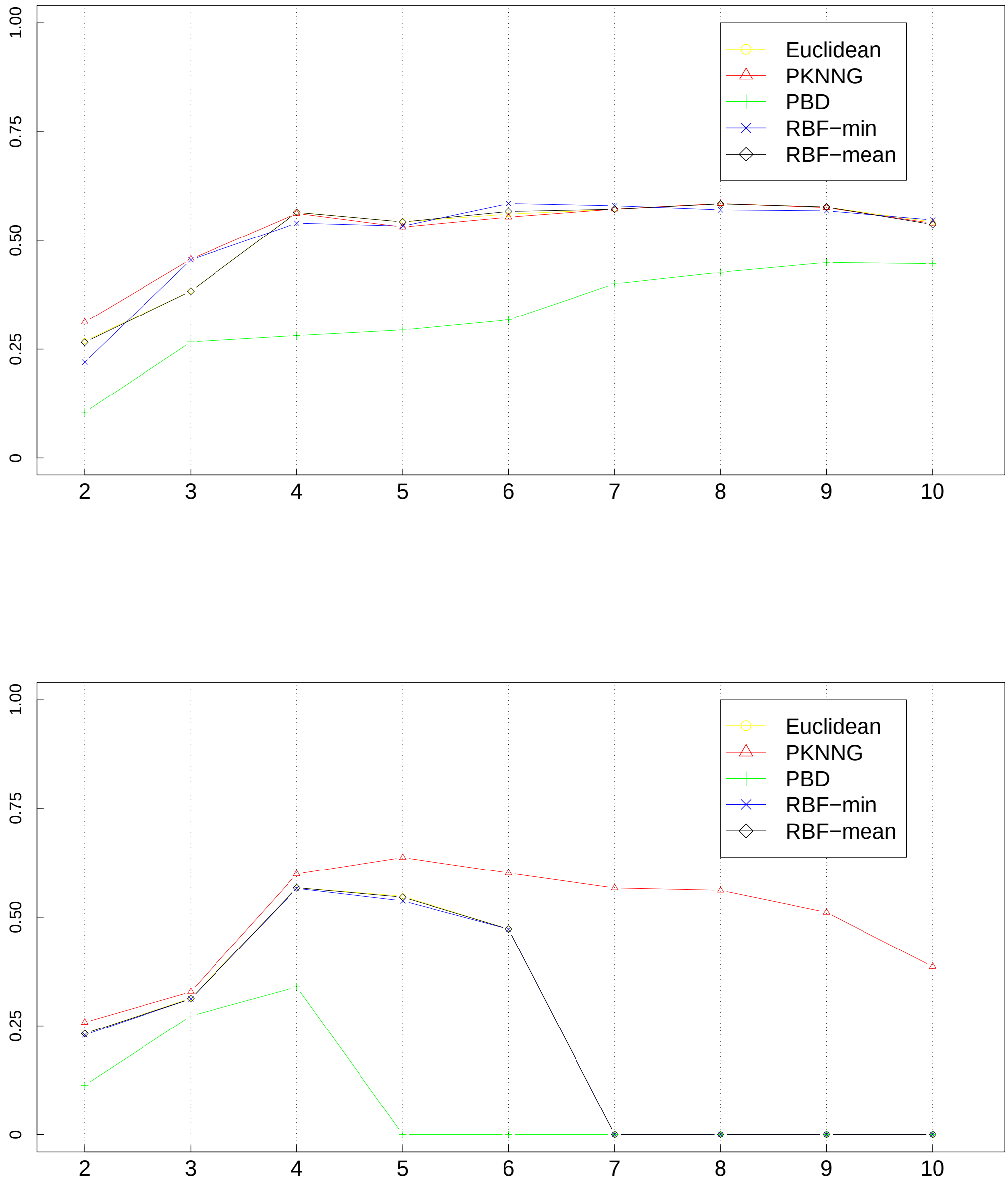

Figure 3: Evaluation of different metrics as a function of the number of clusters extracted for the CNS gene expression dataset (5 classes) using the Euclidean base metric. Top panel: PAM clustering. Bottom panel: HC-av clustering.

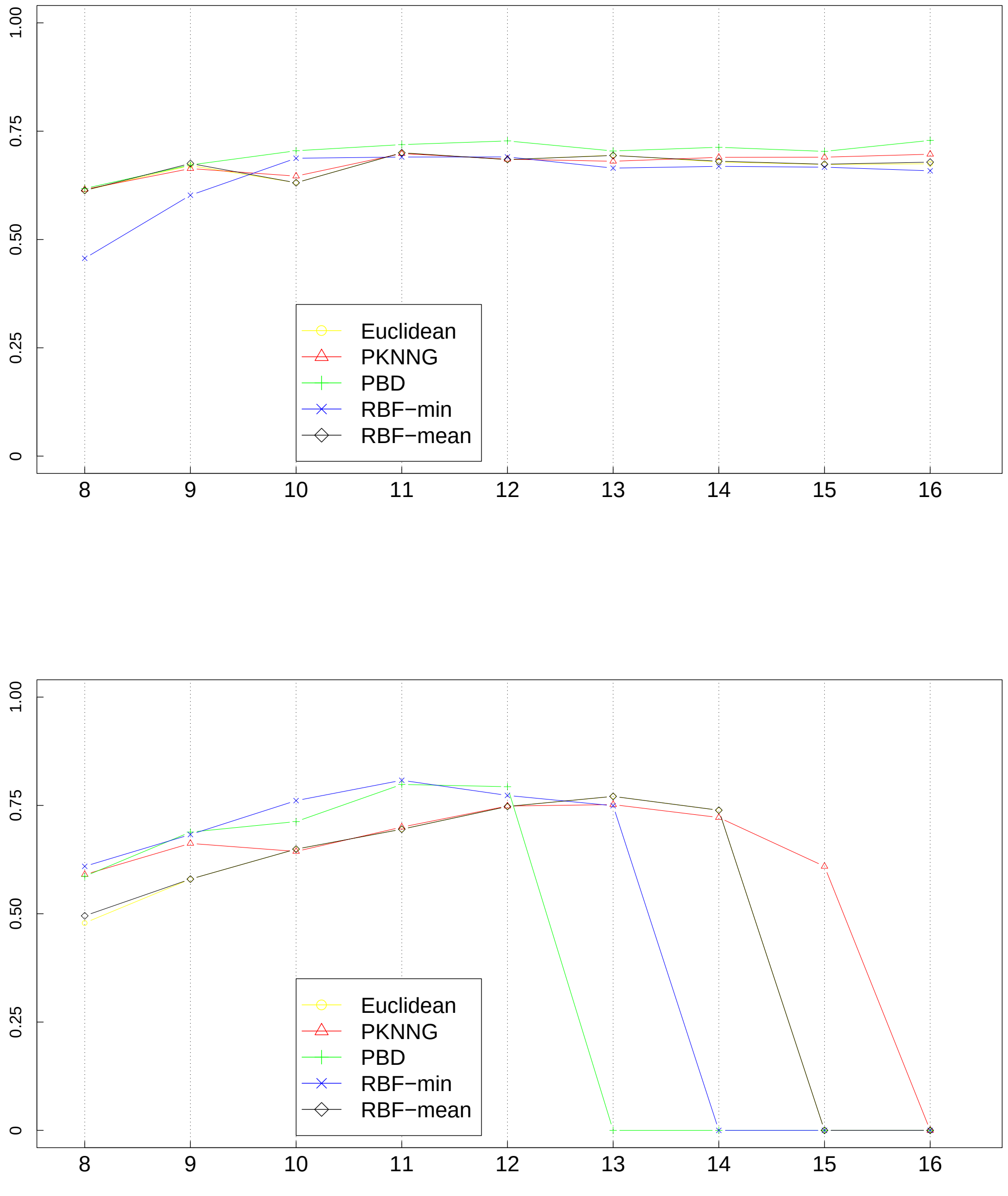

Figure 4: Evaluation of different metrics as a function of the number of clusters extracted for the CGM gene expression dataset (13 classes) using the Euclidean base metric. Top panel: PAM clustering. Bottom panel: HC-av clustering.

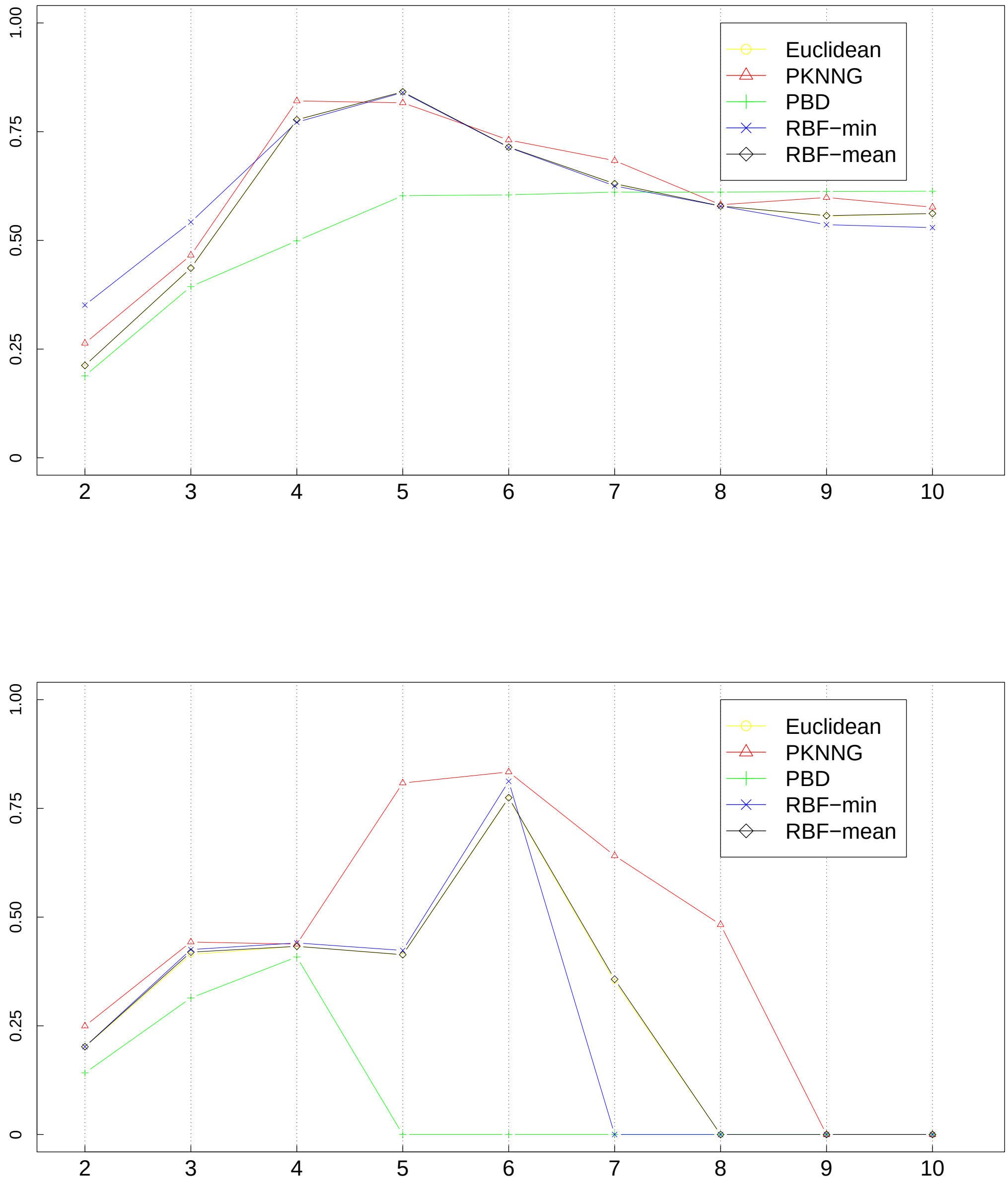

Figure 5: Evaluation of different metrics as a function of the number of clusters extracted for the LEU gene expression dataset (6 classes) using the Euclidean base metric. Top panel: PAM clustering. Bottom panel: HC-av clustering.

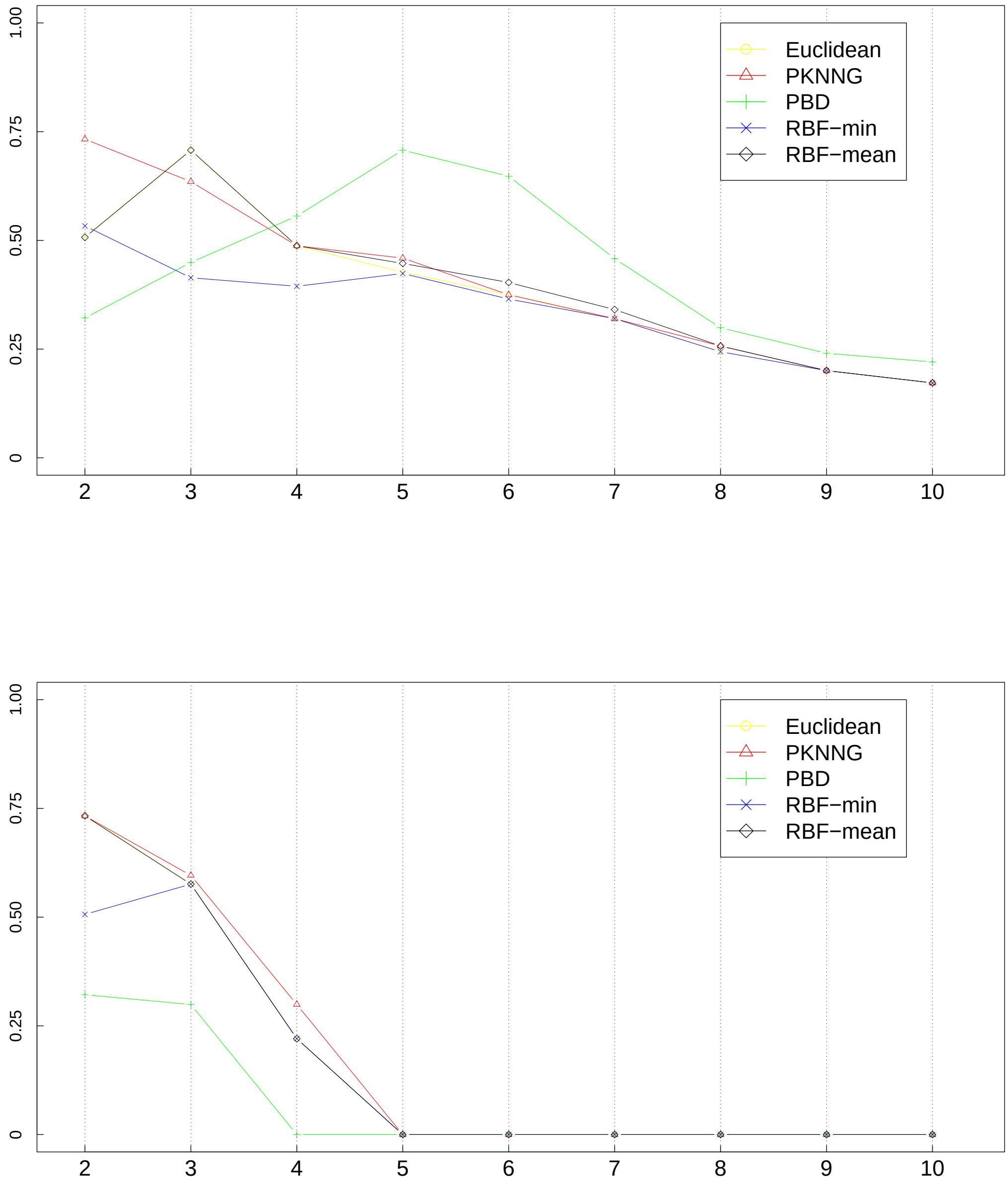

Figure 6: Evaluation of different metrics as a function of the number of clusters extracted for the THY gene expression dataset (2 classes) using the Euclidean base metric. Top panel: PAM clustering. Bottom panel: HC-av clustering.

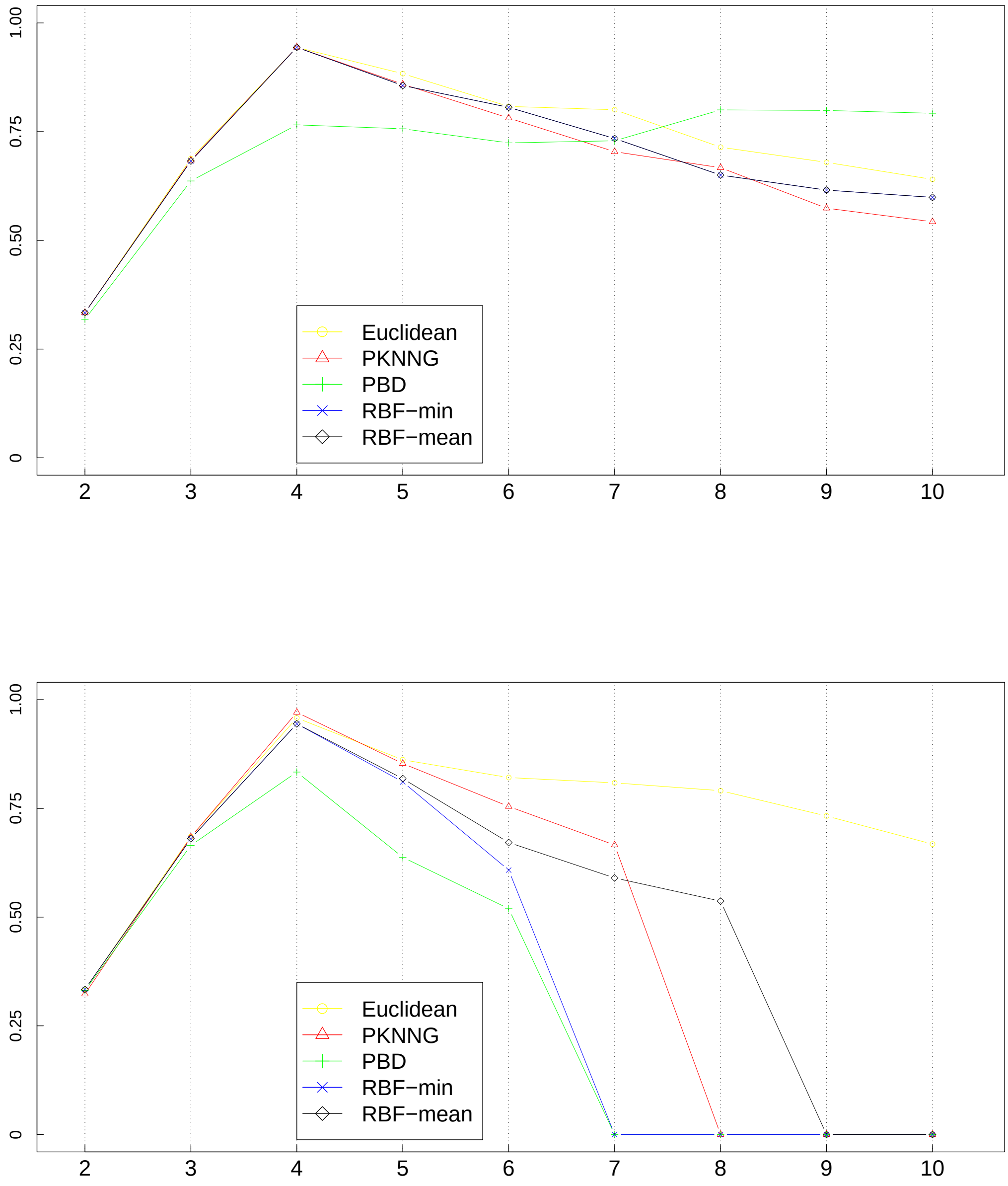

Figure 7: Evaluation of different metrics as a function of the number of clusters extracted for the BCLP gene expression dataset (4 classes) using the Euclidean base metric. Top panel: PAM clustering. Bottom panel: HC-av clustering.

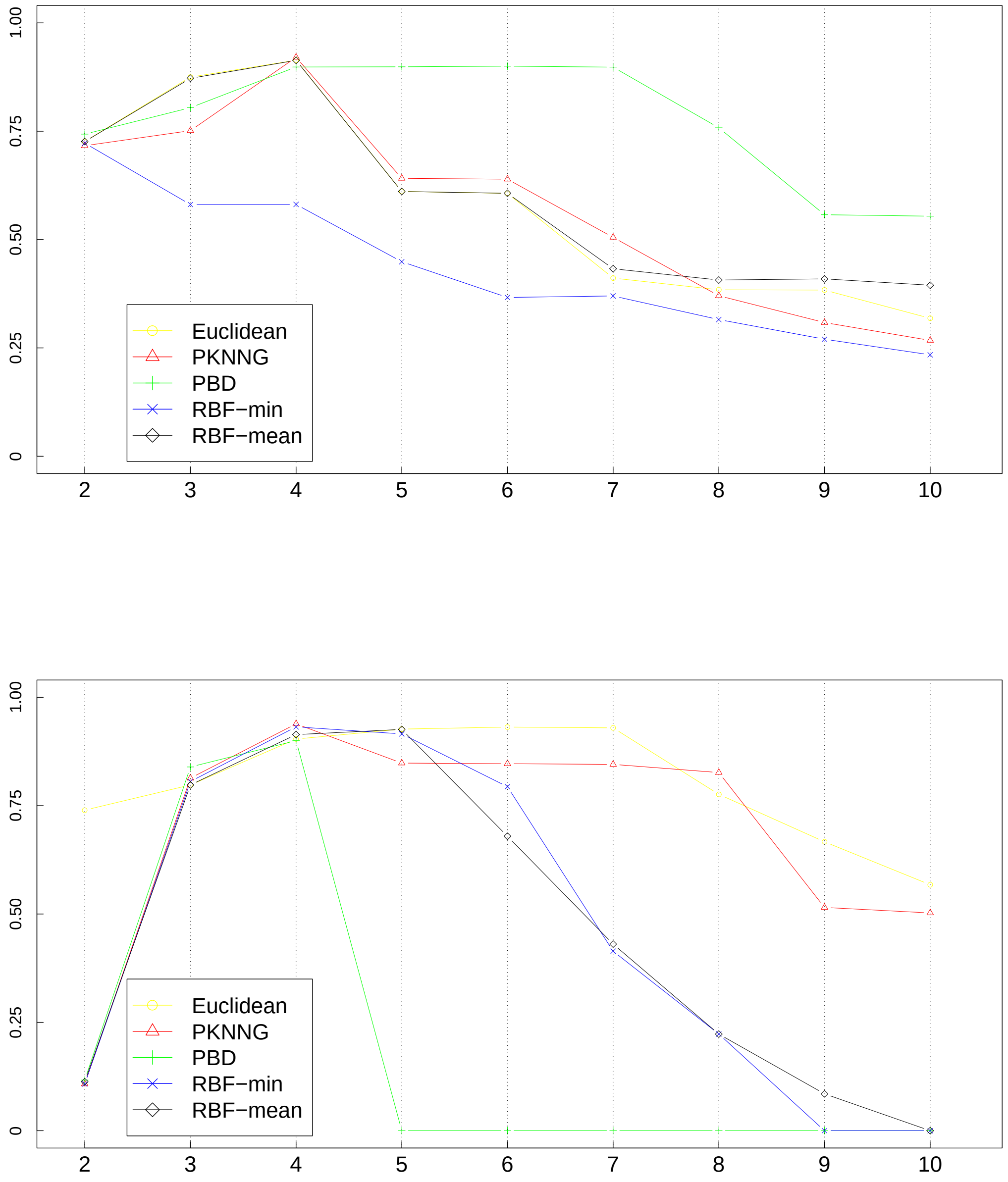

Figure 8: Evaluation of different metrics as a function of the number of clusters extracted for the Y gene expression dataset (4 classes) using the Euclidean base metric. Top panel: PAM clustering. Bottom panel: HC-av clustering.

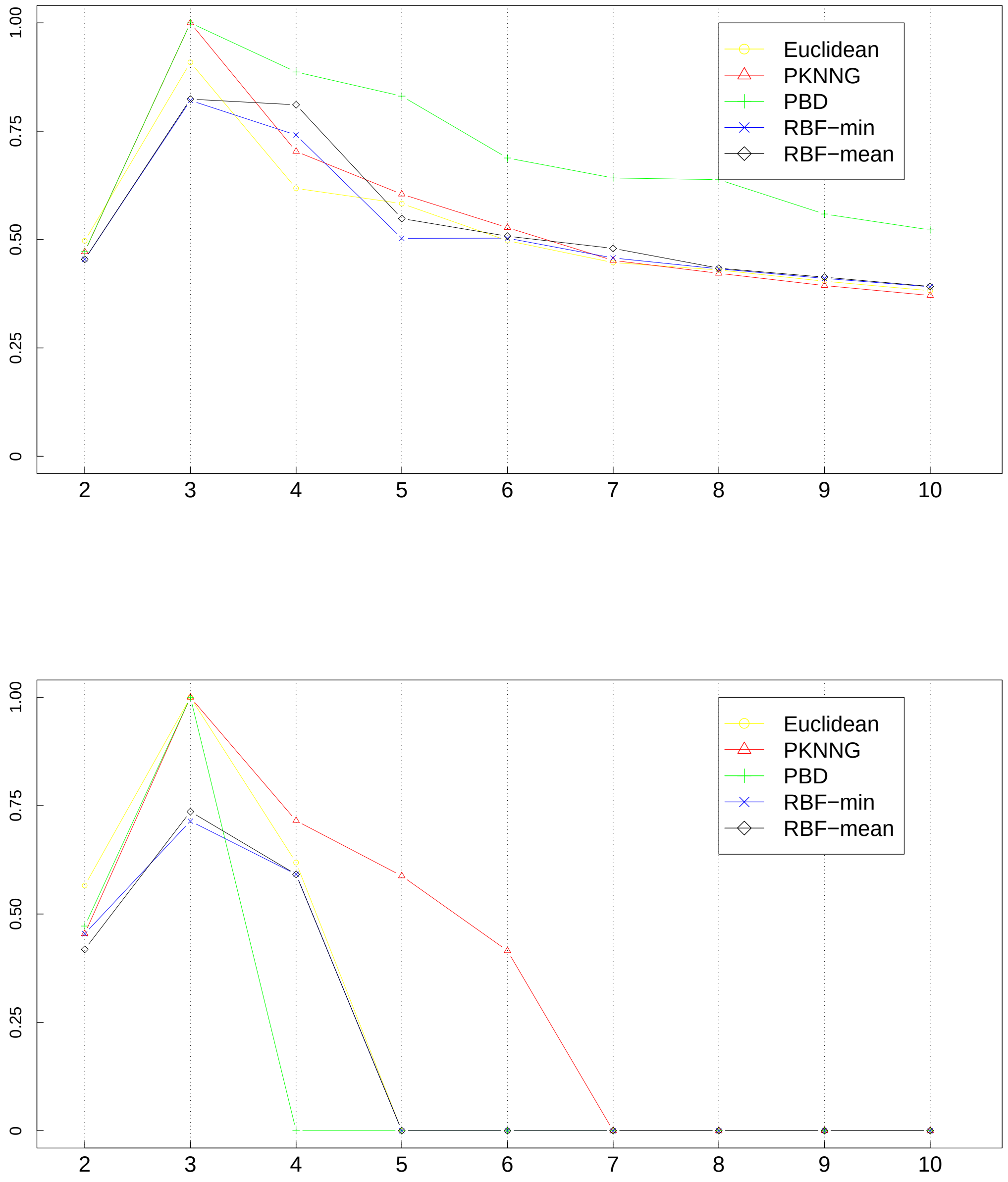

Figure 9: Evaluation of different metrics as a function of the number of clusters extracted for the ALB gene expression dataset (3 classes) using Pearson's correlation as base metric. Top panel: PAM clustering. Bottom panel: HC-av clustering.

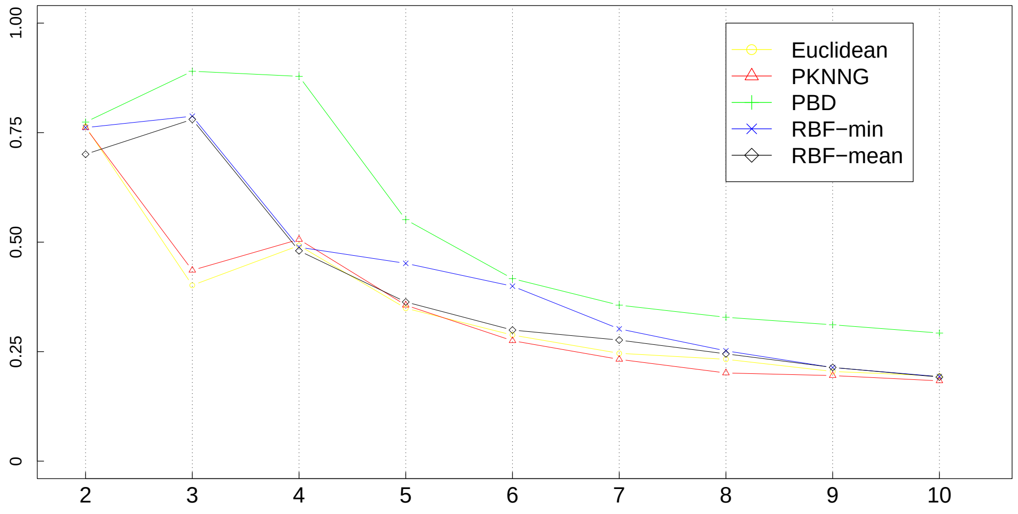

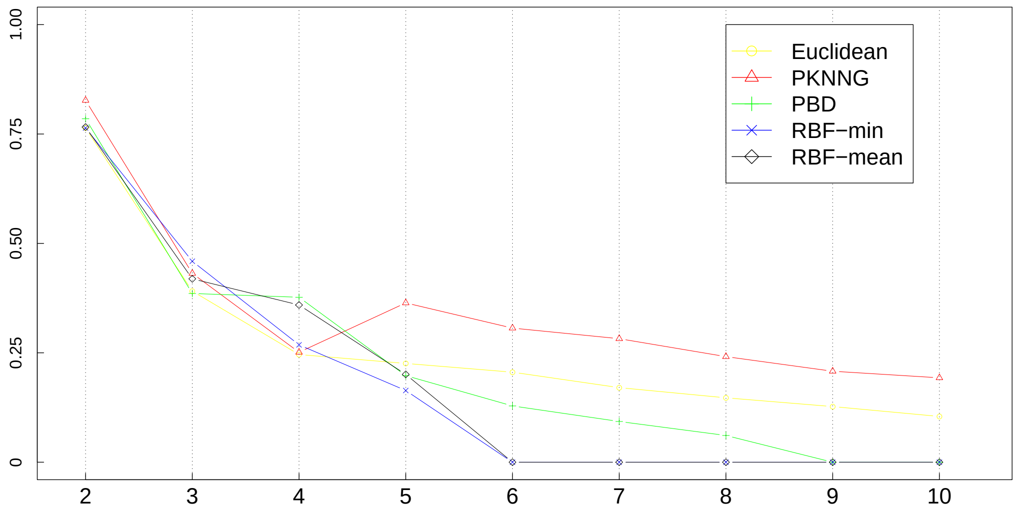

Figure 10: Evaluation of different metrics as a function of the number of clusters extracted for the ALI gene expression dataset (3 classes) using Pearson's correlation as base metric. Top panel: PAM clustering. Bottom panel: HC-av clustering.

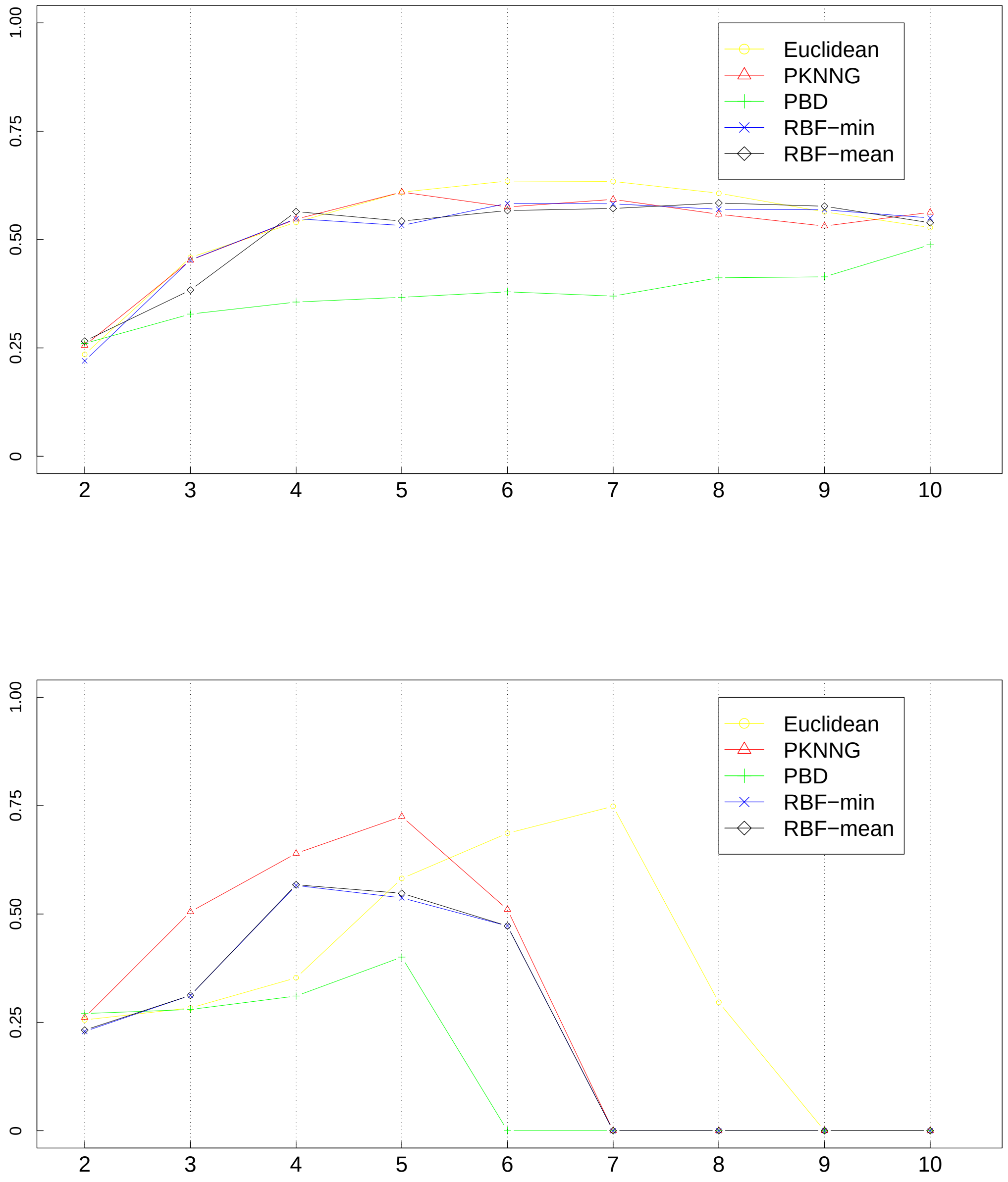

Figure 11: Evaluation of different metrics as a function of the number of clusters extracted for the CNS gene expression dataset (5 classes) using Pearson's correlation as base metric. Top panel: PAM clustering. Bottom panel: HC-av clustering.

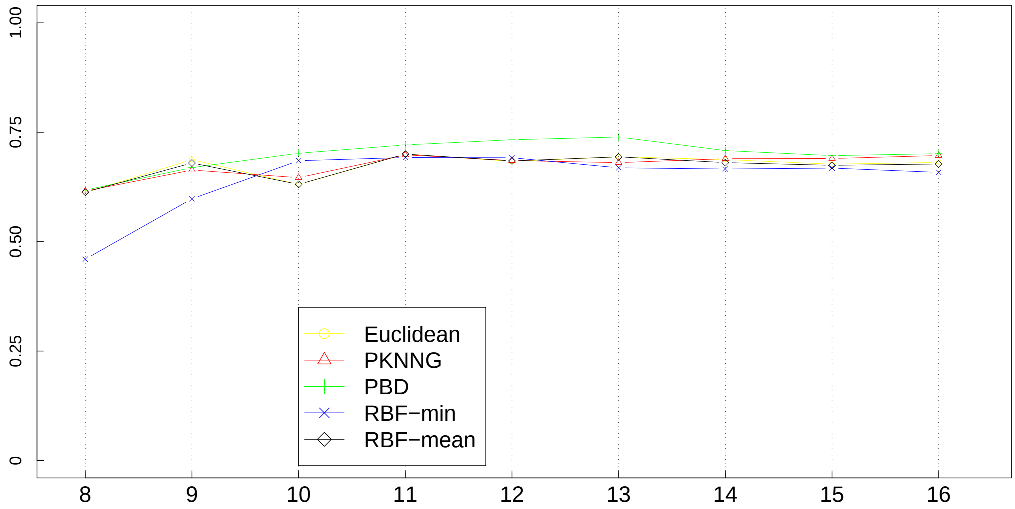

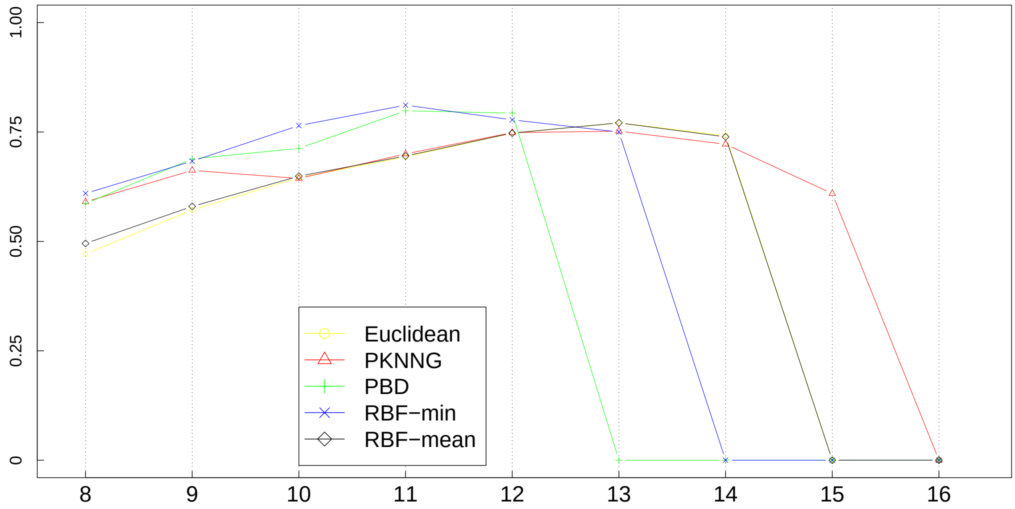

Figure 12: Evaluation of different metrics as a function of the number of clusters extracted for the CGM gene expression dataset (13 classes) using Pearson's correlation as base metric. Top panel: PAM clustering. Bottom panel: HC-av clustering.

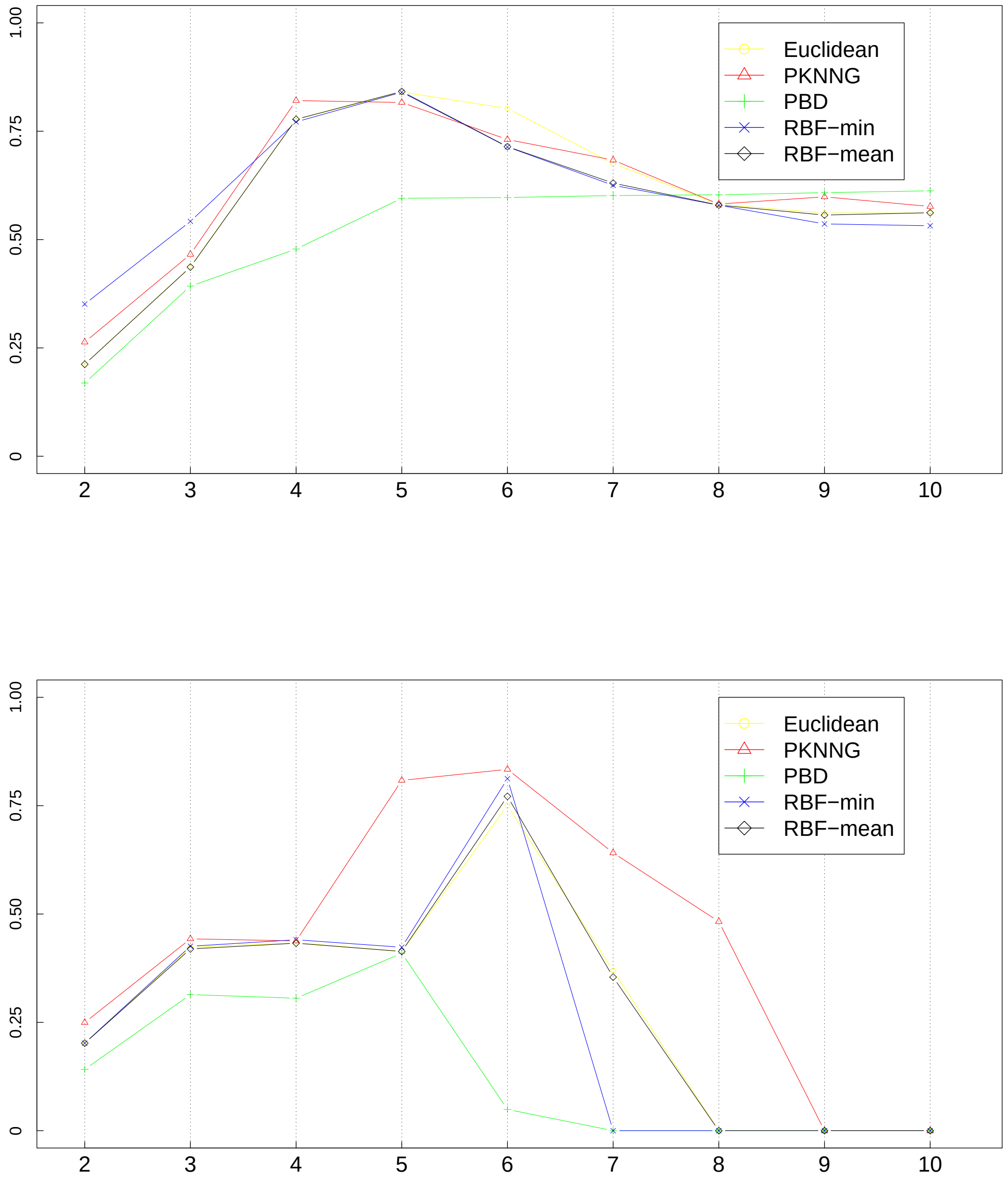

Figure 13: Evaluation of different metrics as a function of the number of clusters extracted for the LEU gene expression dataset (6 classes) using Pearson's correlation as base metric. Top panel: PAM clustering. Bottom panel: HC-av clustering.

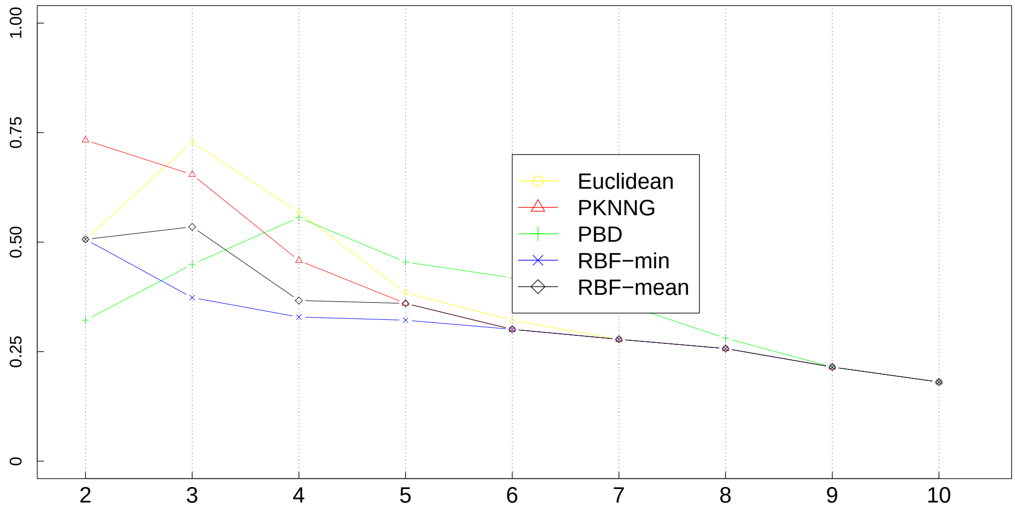

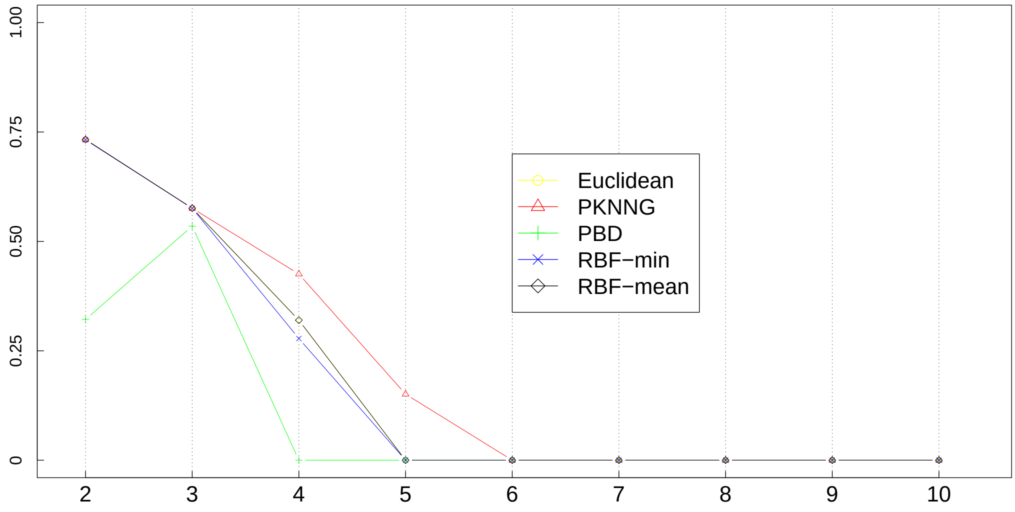

Figure 14: Evaluation of different metrics as a function of the number of clusters extracted for the THY gene expression dataset (2 classes) using Pearson's correlation as base metric. Top panel: PAM clustering. Bottom panel: HC-av clustering.

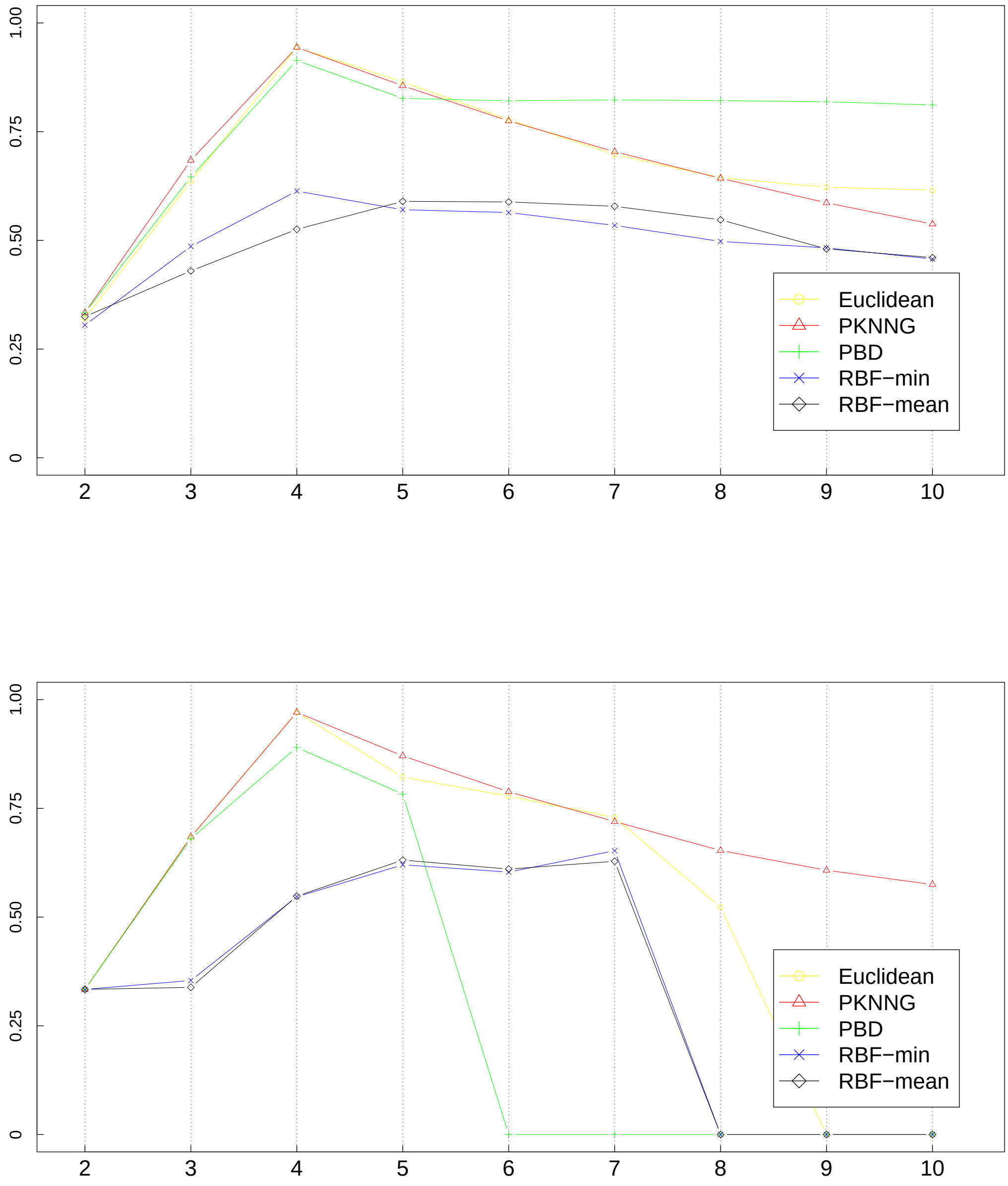

Figure 15: Evaluation of different metrics as a function of the number of clusters extracted for the BCLP gene expression dataset (4 classes) using Pearson's correlation as base metric. Top panel: PAM clustering. Bottom panel: HC-av clustering.

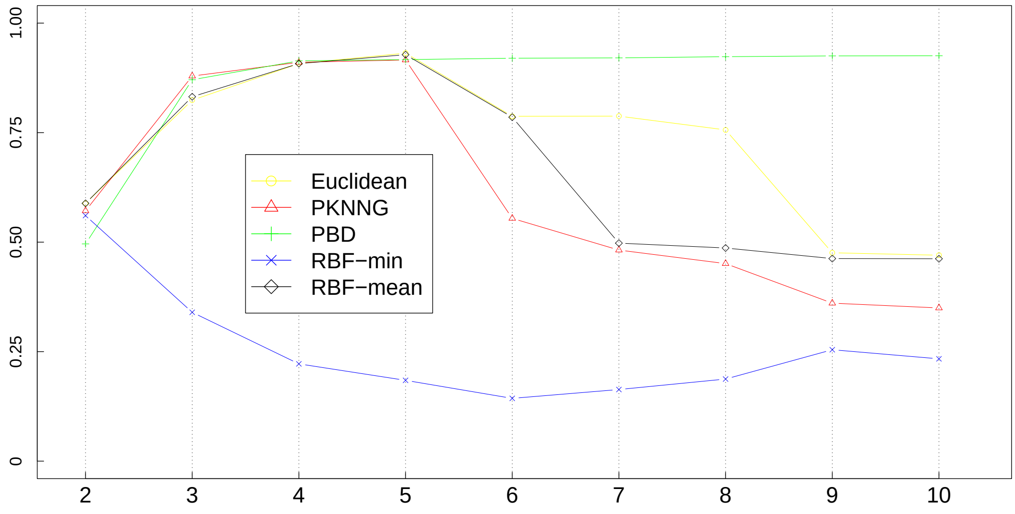

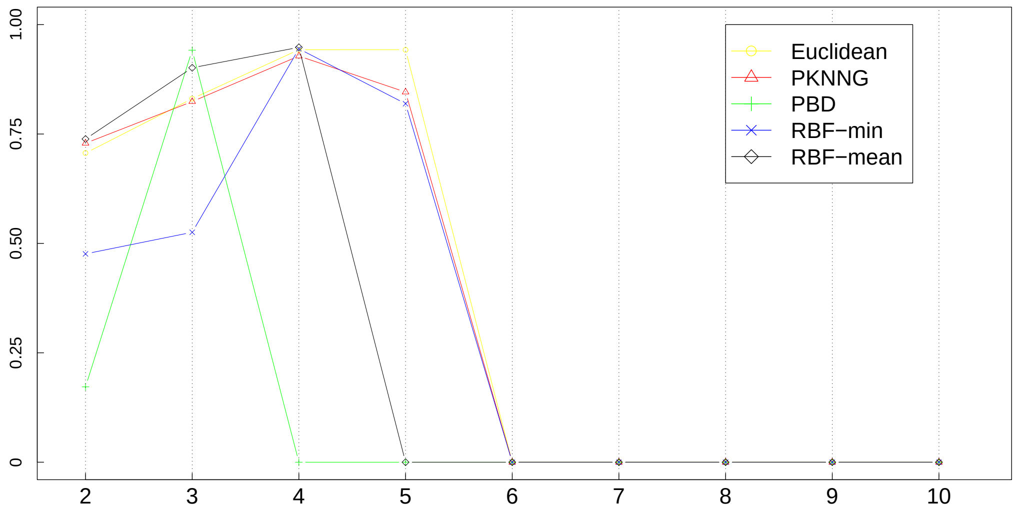

Figure 16: Evaluation of different metrics as a function of the number of clusters extracted for the Y gene expression dataset (4 classes) using Pearson's correlation as base metric. Top panel: PAM clustering. Bottom panel: HC-av clustering.

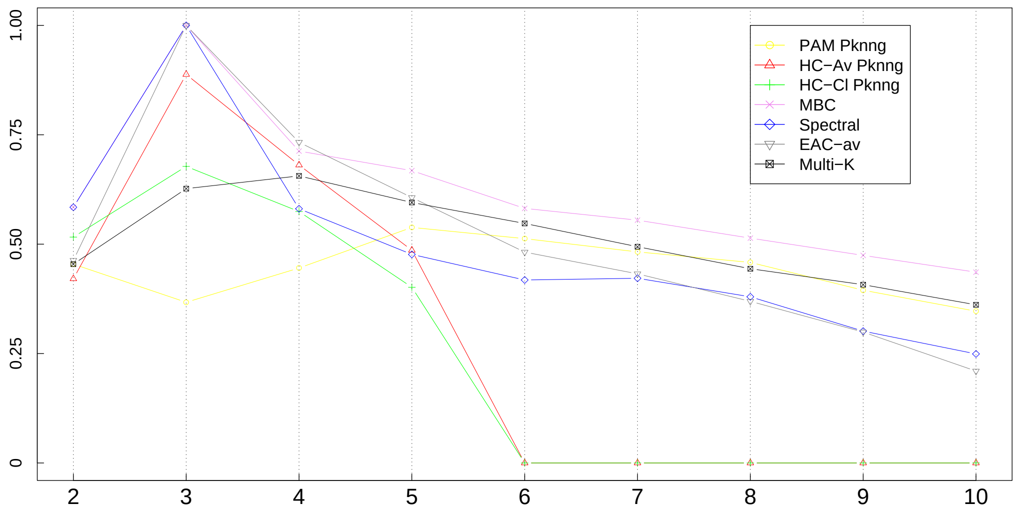

Figure 17: Evaluation of different clustering methods as a function of the number of clusters extracted for the ALB gene expression dataset (3 classes) using the Euclidean base metric.

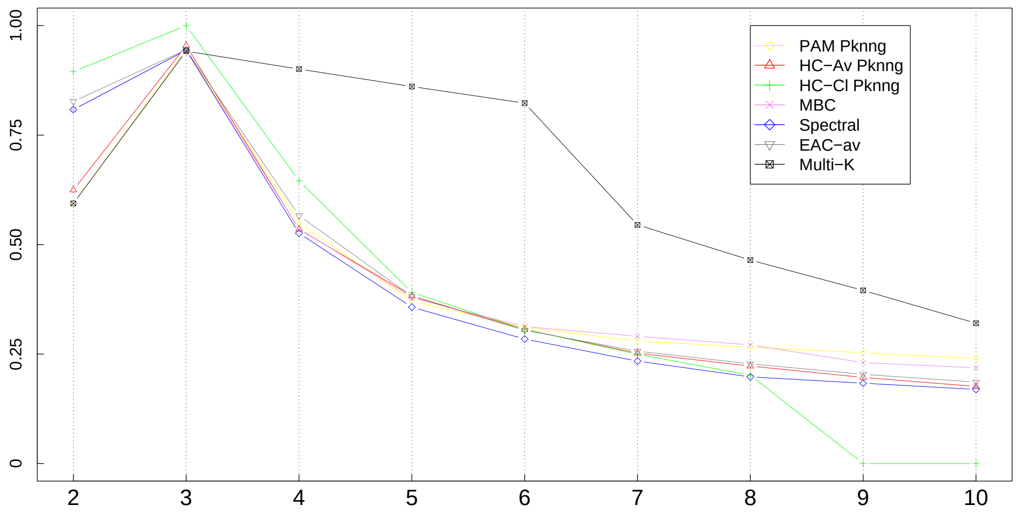

Figure 18: Evaluation of different clustering methods as a function of the number of clusters extracted for the ALI gene expression dataset (3 classes) using the Euclidean base metric.

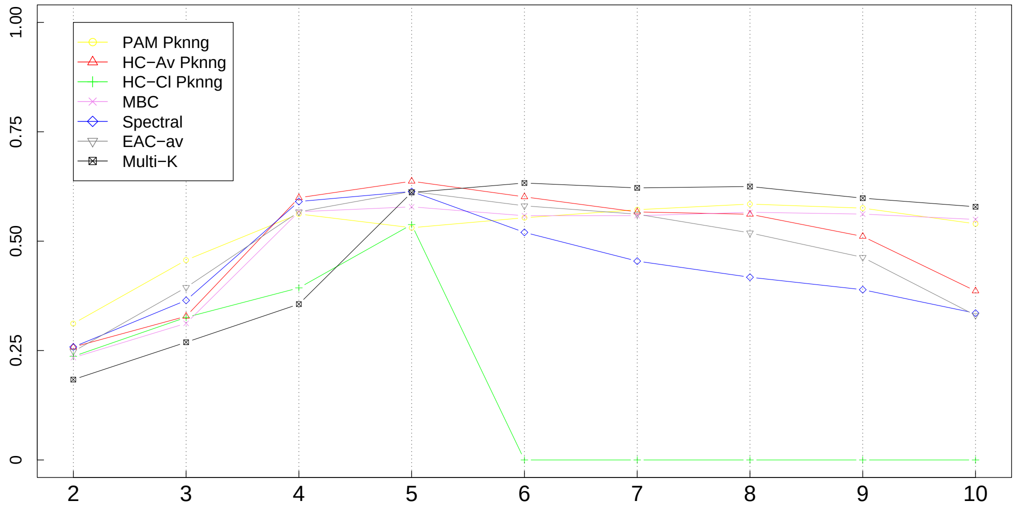

Figure 19: Evaluation of different clustering methods as a function of the number of clusters extracted for the CNS gene expression dataset (5 classes) using the Euclidean base metric.

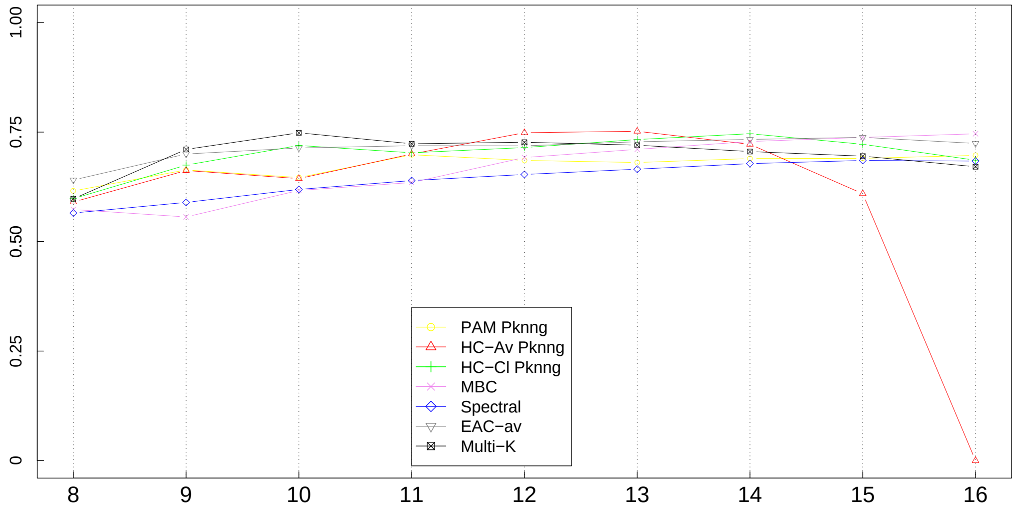

Figure 20: Evaluation of different clustering methods as a function of the number of clusters extracted for the CGM gene expression dataset (13 classes) using the Euclidean base metric.

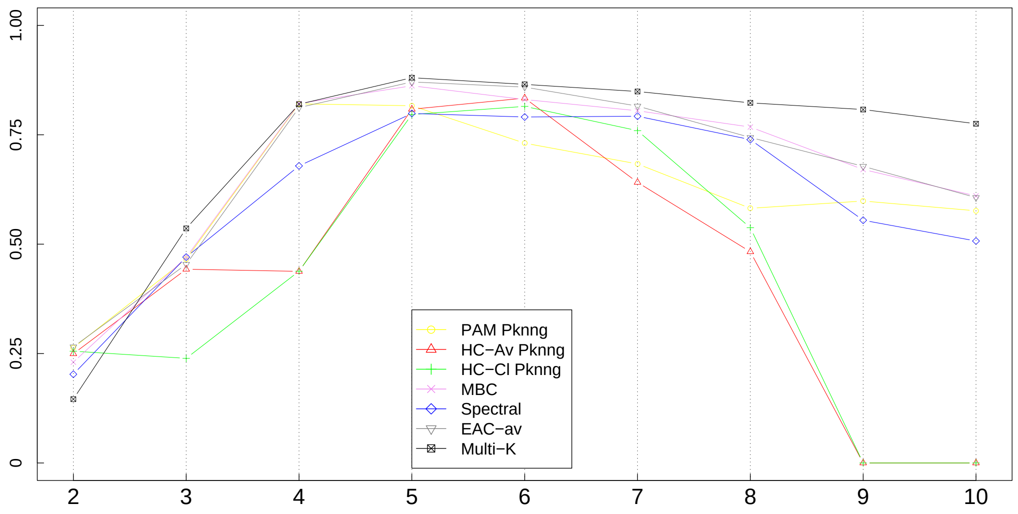

Figure 21: Evaluation of different clustering methods as a function of the number of clusters extracted for the LEU gene expression dataset (6 classes) using the Euclidean base metric.

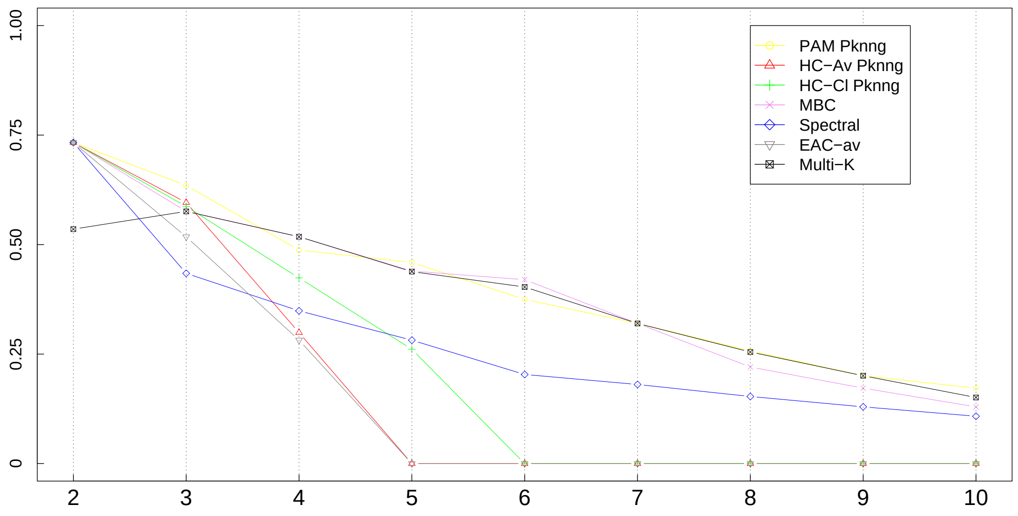

Figure 22: Evaluation of different clustering methods as a function of the number of clusters extracted for the THY gene expression dataset (2 classes) using the Euclidean base metric.

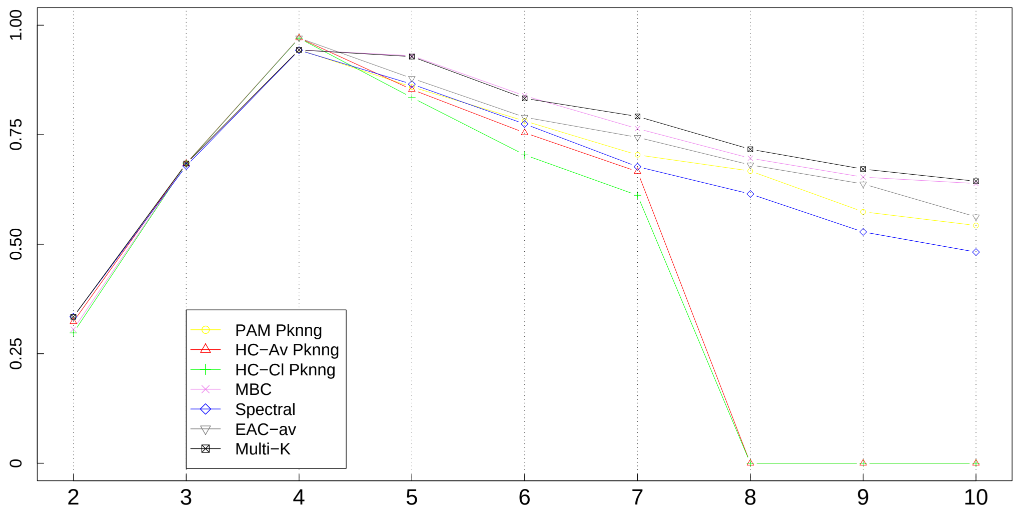

Figure 23: Evaluation of different clustering methods as a function of the number of clusters extracted for the BCLP gene expression dataset (4 classes) using the Euclidean base metric.

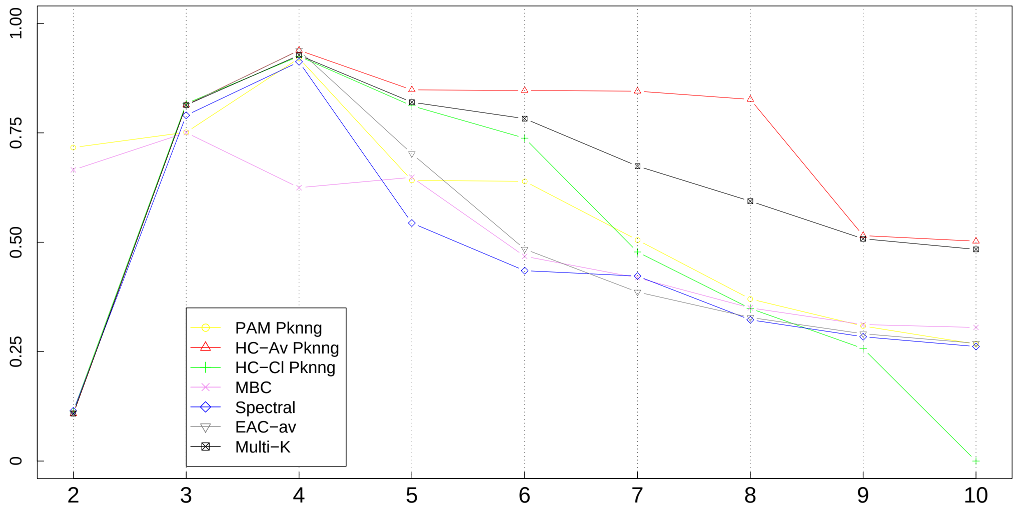

Figure 24: Evaluation of different clustering methods as a function of the number of clusters extracted for the Y gene expression dataset (4 classes) using the Euclidean base metric.

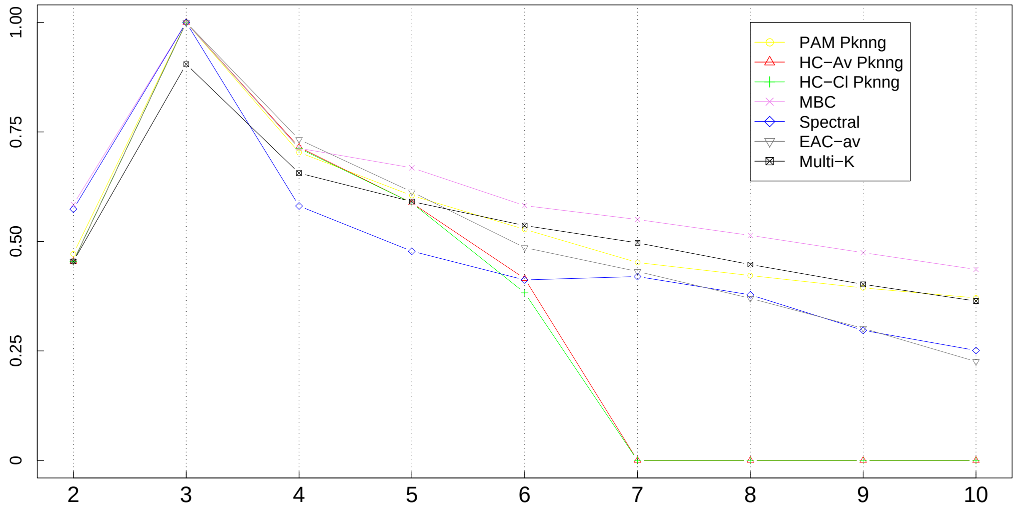

Figure 25: Evaluation of different clustering methods as a function of the number of clusters extracted for the ALB gene expression dataset (3 classes) usingPearson's correlation as base metric.

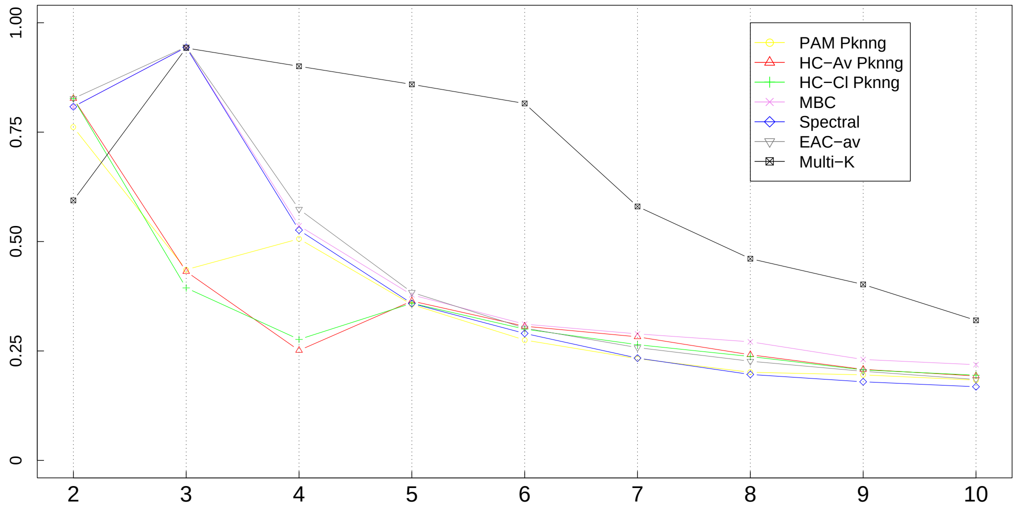

Figure 26: Evaluation of different clustering methods as a function of the number of clusters extracted for the ALI gene expression dataset (3 classes) usingPearson's correlation as base metric.

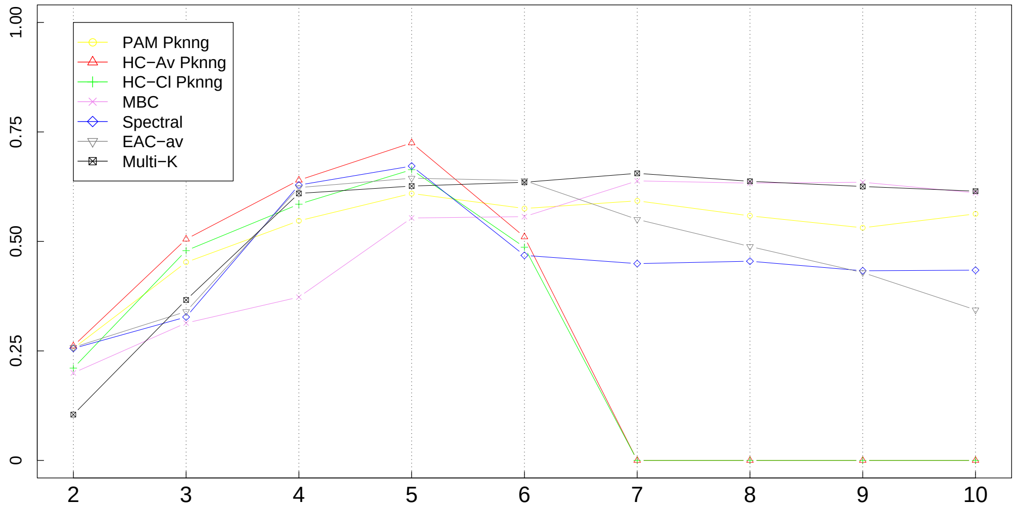

Figure 27: Evaluation of different clustering methods as a function of the number of clusters extracted for the CNS gene expression dataset (5 classes) usingPearson's correlation as base metric.

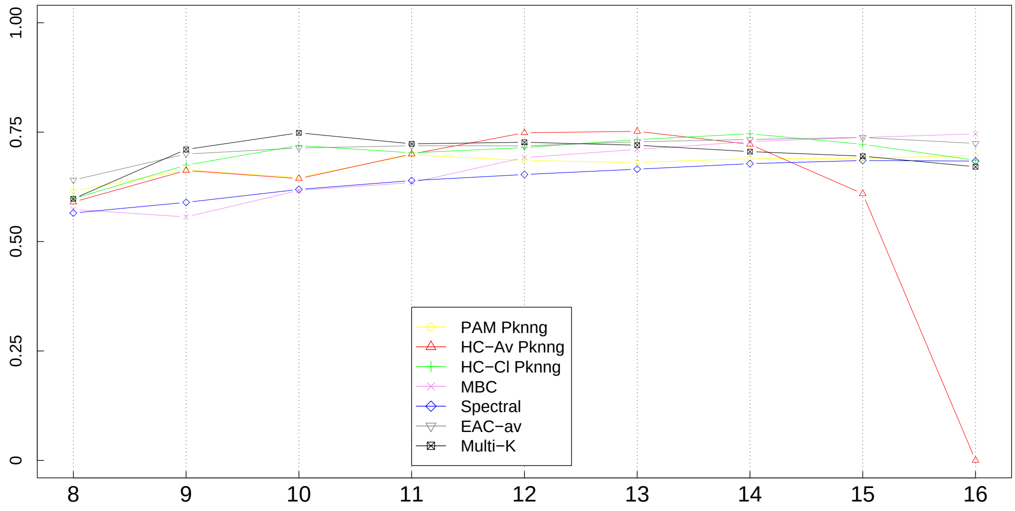

Figure 28: Evaluation of different clustering methods as a function of the number of clusters extracted for the CGM gene expression dataset (13 classes) usingPearson's correlation as base metric.

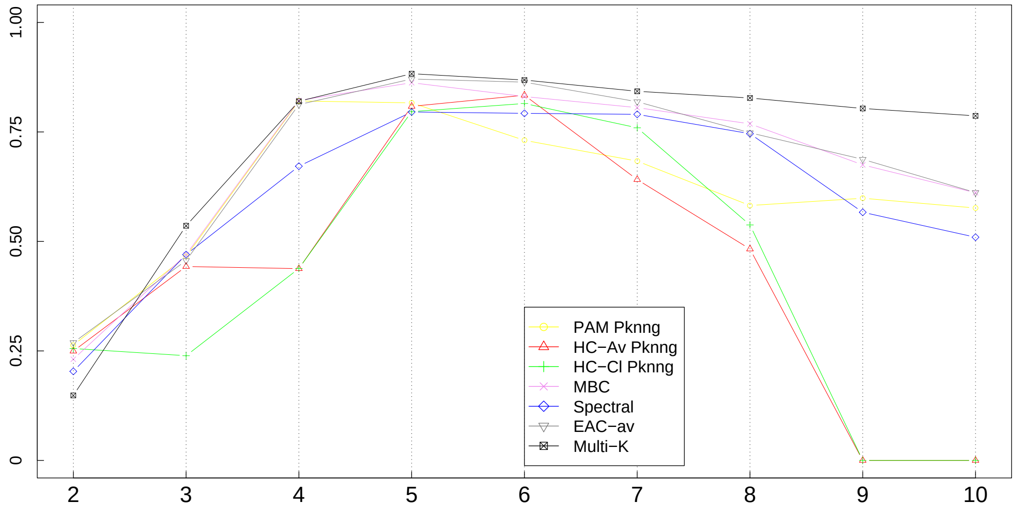

Figure 29: Evaluation of different clustering methods as a function of the number of clusters extracted for the LEU gene expression dataset (6 classes) usingPearson's correlation as base metric.

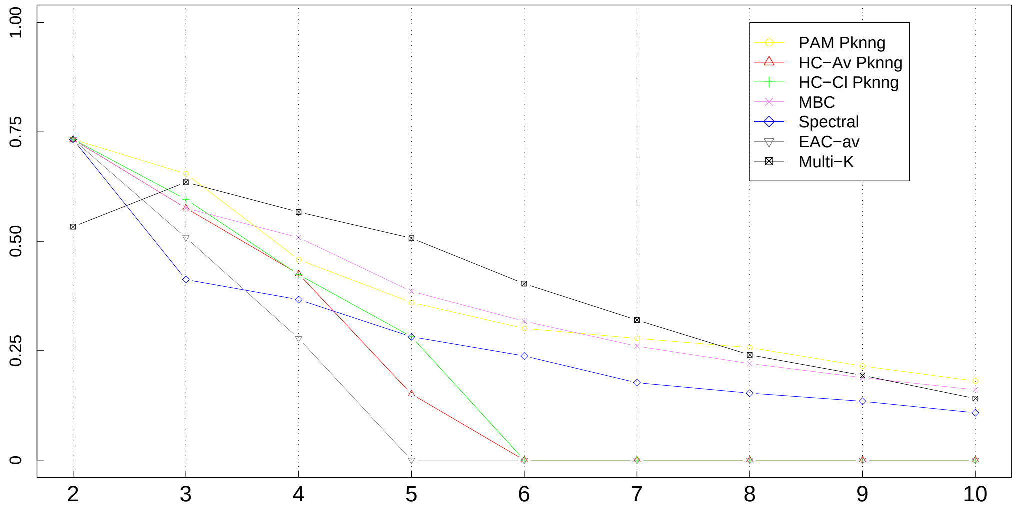

Figure 30: Evaluation of different clustering methods as a function of the number of clusters extracted for the THY gene expression dataset (2 classes) usingPearson's correlation as base metric.

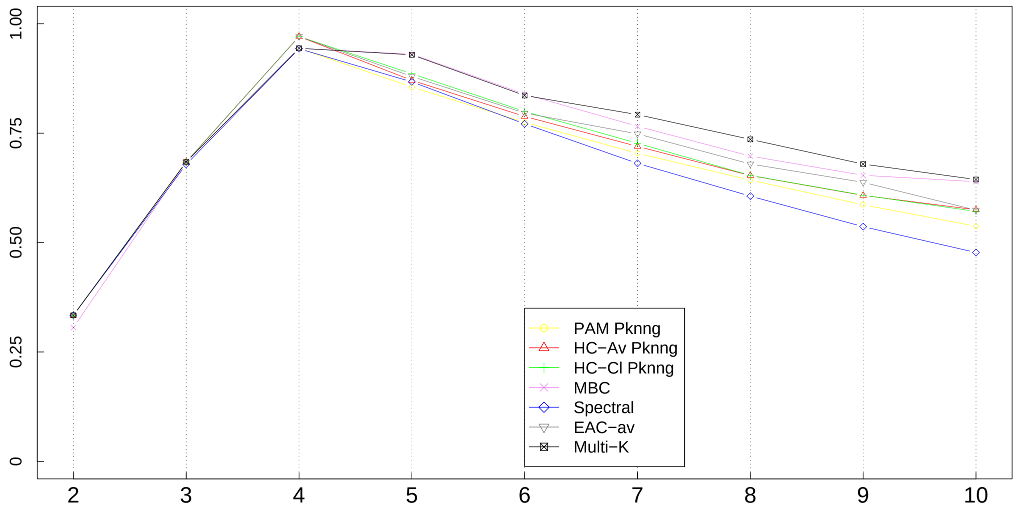

Figure 31: Evaluation of different clustering methods as a function of the number of clusters extracted for the BCLP gene expression dataset (4 classes) usingPearson's correlation as base metric.

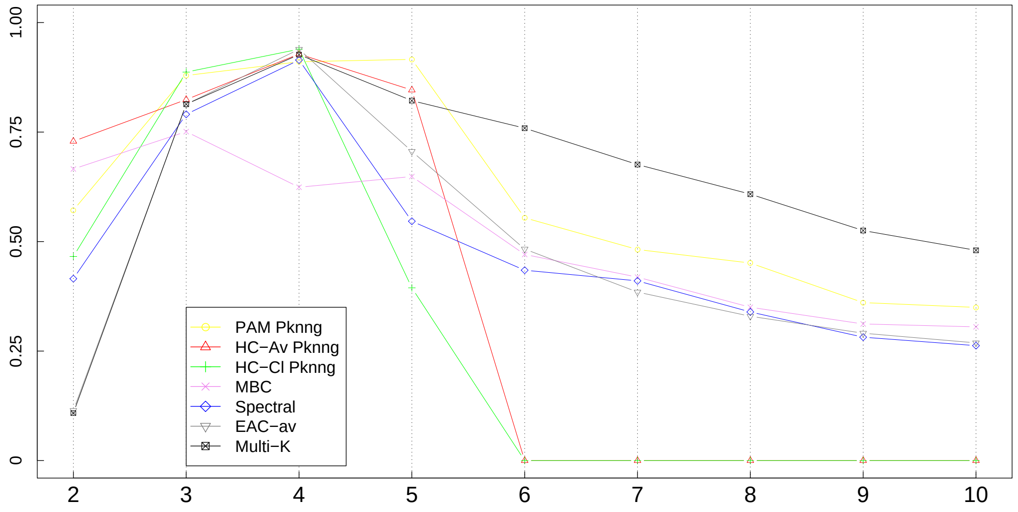

Figure 32: Evaluation of different clustering methods as a function of the number of clusters extracted for the Y gene expression dataset (4 classes) usingPearson's correlation as base metric.
