# Supplementary material for: Clustering gene expression data with a penalized graph-based metric
Source: BMC Bioinformatics. 2011 Jan 4;12:2. doi: 10.1186/1471-2105-12-2 (PMC3023695; doi:10.1186/1471-2105-12-2)
Supplement: Additional file 3 — Full Figures. Complete versions of Figure 15 and 16, including the four embeddings discussed in the Methods section. [file 1471-2105-12-2-S3.PDF]

Clustering gene expression data with a penalized graph-based metric

A. Baya & P.M. Granitto

Full Figures

This file includes the complete versions of Figures 15 and 16 in the paper. The arrangement of each figure is the same as in the paper: Columns of sub-figures correspond, from left to right, to the Two-moons, Three-spirals and Three-rings datasets (introduced in the paper). Rows correspond to the four different embeddings; from top to bottom: 2d, 3d, 3d–noise and 10d–noise. Each sub-figure shows the mean clustering accuracy of the diverse methods at three different noise levels.

We also include an additional figure in which we compare three clustering methods (PAM, HC and AP) that we described in the Introduction, on the same artificial problems, in order to check that there is not any fundamental dependence of the performance of the PKNNG metric on the clustering algorithm being used.

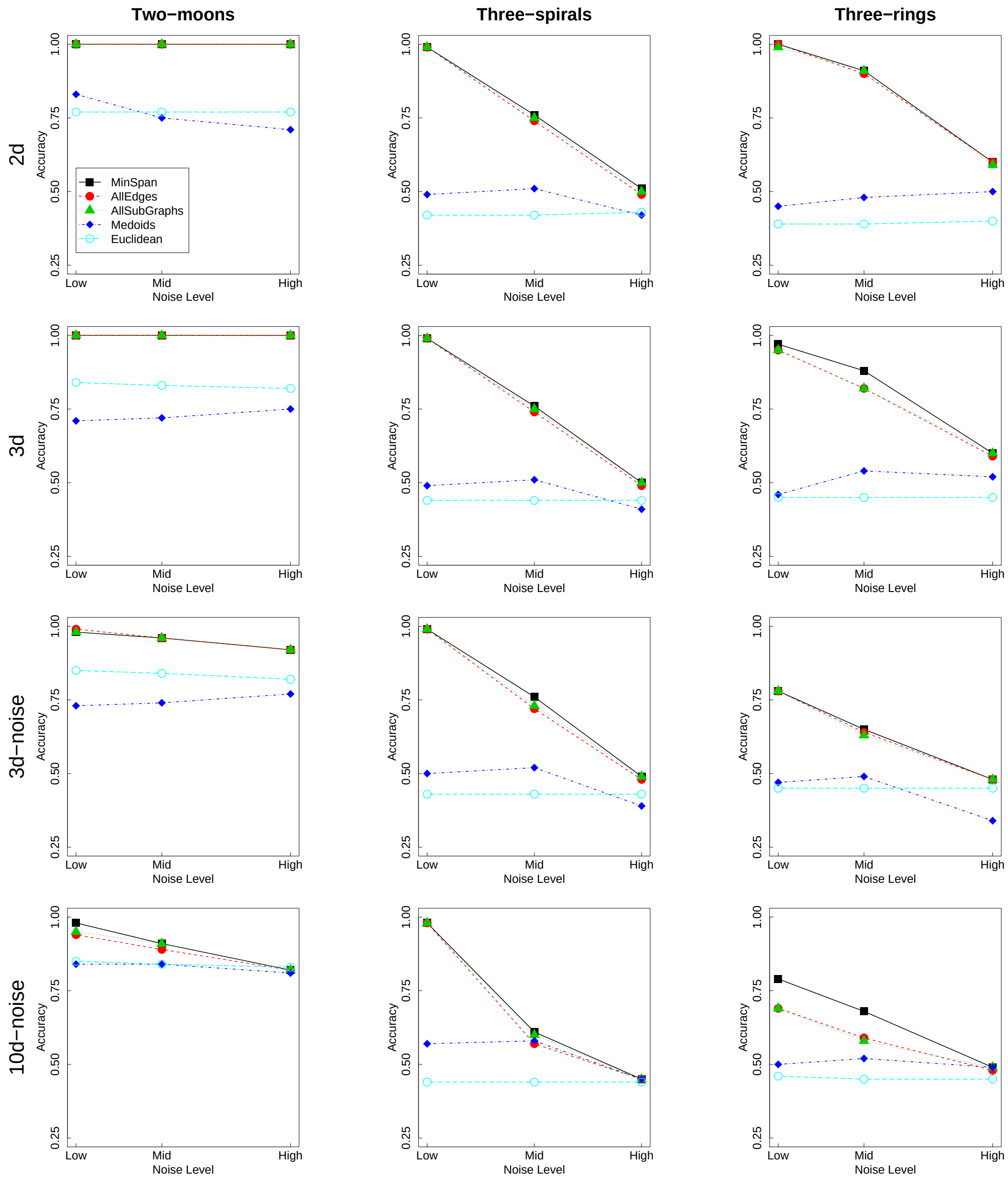

Figure 1: Comparison of the different connection schemes (the full version of Figure 15 in the paper).

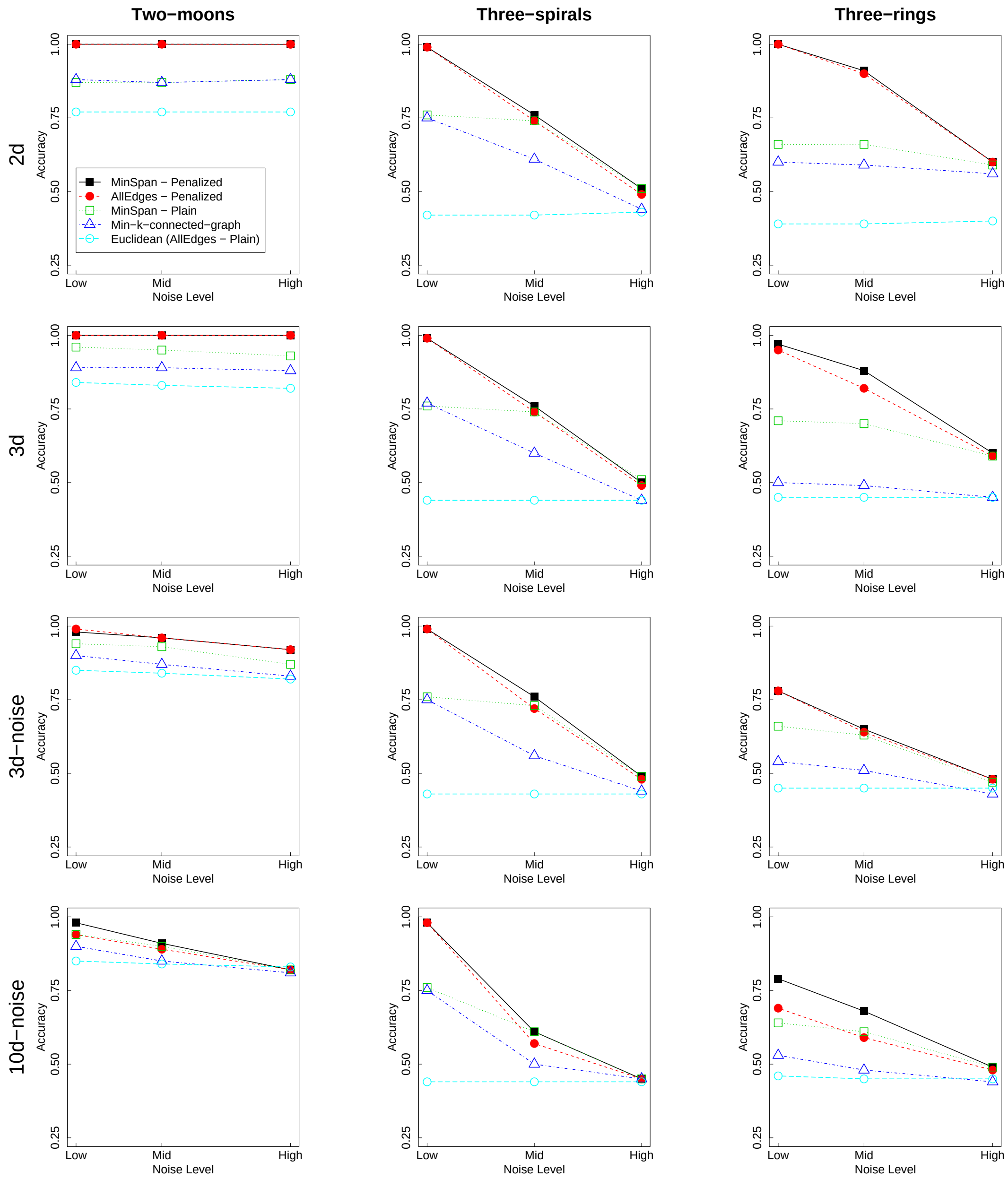

Figure 2: Comparison of two connection schemes (MinSpan and AllEdges) in their "plain" and exponentially penalized forms (the full version of Figure 16 in the paper).

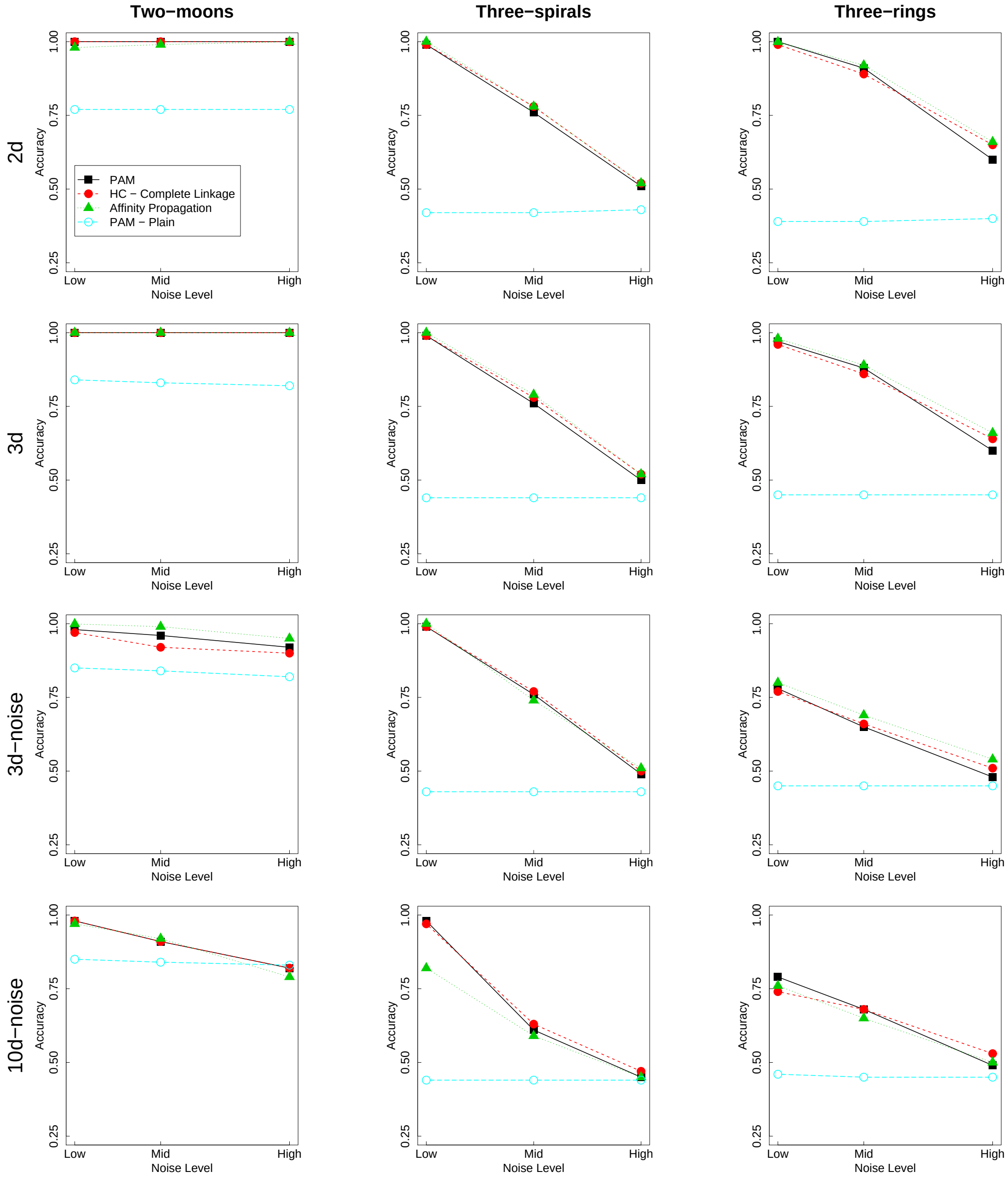

Figure 3: Additional Figure: Comparison of three different clustering algorithms applied to similarities evaluated with the PKNNG (MinSpan-Exponential) metric.
