# Supplementary material for: Clustering gene expression data with a penalized graph-based metric
Source: BMC Bioinformatics. 2011 Jan 4;12:2. doi: 10.1186/1471-2105-12-2 (PMC3023695; doi:10.1186/1471-2105-12-2)
Supplement: Additional file 4 — Evaluation of Penalization Functions. Discussion and evaluation of other penalization functions. [file 1471-2105-12-2-S4.PDF]

Clustering gene expression data with a penalized graph-based metric
A. Baya & P.M. Granitto
Evaluation of Penalization Functions

This file includes a series of figures evaluating different penalization functions for the PKNNG metric.

Unless stated otherwise, the arrangement of each figure in this section is the following: Columns of sub-figures correspond, from left to right, to the Two-moons, Three-spirals and Three-rings datasets (introduced in the paper). Rows correspond to the four different embeddings; from top to bottom: 2d, 3d, 3d–noise and 10d–noise. Each sub-figure shows the mean clustering accuracy of the diverse methods at three different noise levels.

In all figures we repeated the results of two penalization in order to have a common base to make comparisons. In all cases we show in black squares, the results of using no penalization for the added edges (identified as "no pen" in all figures) and in red circles the results of the exponential penalization that we used in the paper (identified as "exp" in all figures).

In Figure 1 we compare two exponentially penalized functions. First, the base exponential function defined and used in the paper: $w = d \ \ e^{d/\mu}$, which is labeled "exp" in the figure, where w is the graph weight corresponding to the added edge, d is the Euclidean distance between the points being connected by that edge and μ is the mean edge weight in the original graph. Second, the factor $w = d \ \ e^{d/\mu - 1}$, labeled "exp-1", which seems more natural as it penalizes only external connections bigger than μ. From the figure it is clear that both penalizations produce very similar results, always better than the base no-penalization method, being the "exp" function slightly more accurate.

In Figure 2 we evaluate a simple power penalization. We consider functions of the form: ($w = d \ (d/\mu)^k$, with $k \in N^+$), where again w is the graph weight corresponding to the added edge, d is the Euclidean distance between the points being connected by that edge and μ is the mean edge weight in the original graph. We considered four values of the power k: 1,2,3 and 5. For two datasets, Two-moons and Three-rings, the bigger the power the better the performance, but for the Three-spirals dataset the biggest power produces the worst results, even below no penalization in some cases. Overall, $k = 3$ produces the best results, equivalent to the exponential penalization.

In Figure 3 we show a variant of the power penalization: $w = d \ (d/\mu + 1)^k$. Using this penalization all added weights increase their length. The results are similar to the simple power function in Figure 2.

In Figure 4 we evaluate the linear penalization function: ($w = ad$, with $a \in \Re^+$), where w is the graph weight corresponding to the added edge, d is the Euclidean distance between the points being connected by that edge and a is the coefficient of the linear function. We considered four values of a: 2,3,5 and 10 ($a = 1$ is the base no penalization function). Bigger a values produce better performances, but the exponential function seems to be superior in this case.

Finally, in our last experiment we explore the dependence of the results of the PKNNG metric with the scaling parameter μ. As we explained in the paper, our base definition for μ is to take it as the mean edge weight in the original graph. We also evaluated other three simple definitions of μ, always referred to the distribution of edge weights in the original graph: the median and the first and third quartiles. In Figure 5 we show the results of using the exponential penalization with these four μ values. The mean, median and first quartile values produce almost the same results. Using the third quartile produces some small differences with the other values, but the results depends on the problem at hand.

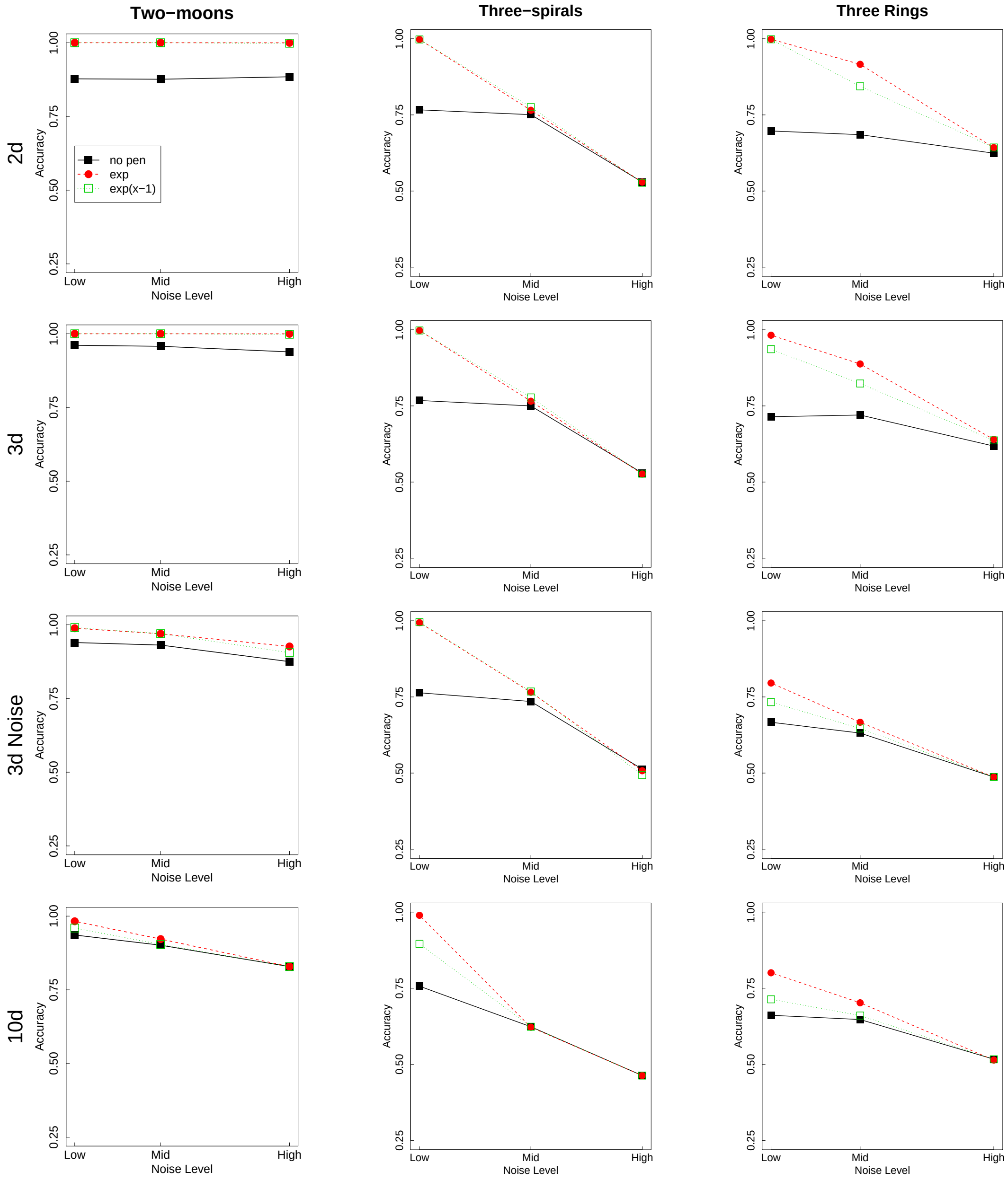

Figure 1: Comparison of two exponential penalization functions: $w = d\ \ e^{d/\mu}$ (red circles) and $w = d\ \ e^{d/\mu - 1}$ (open green squares).

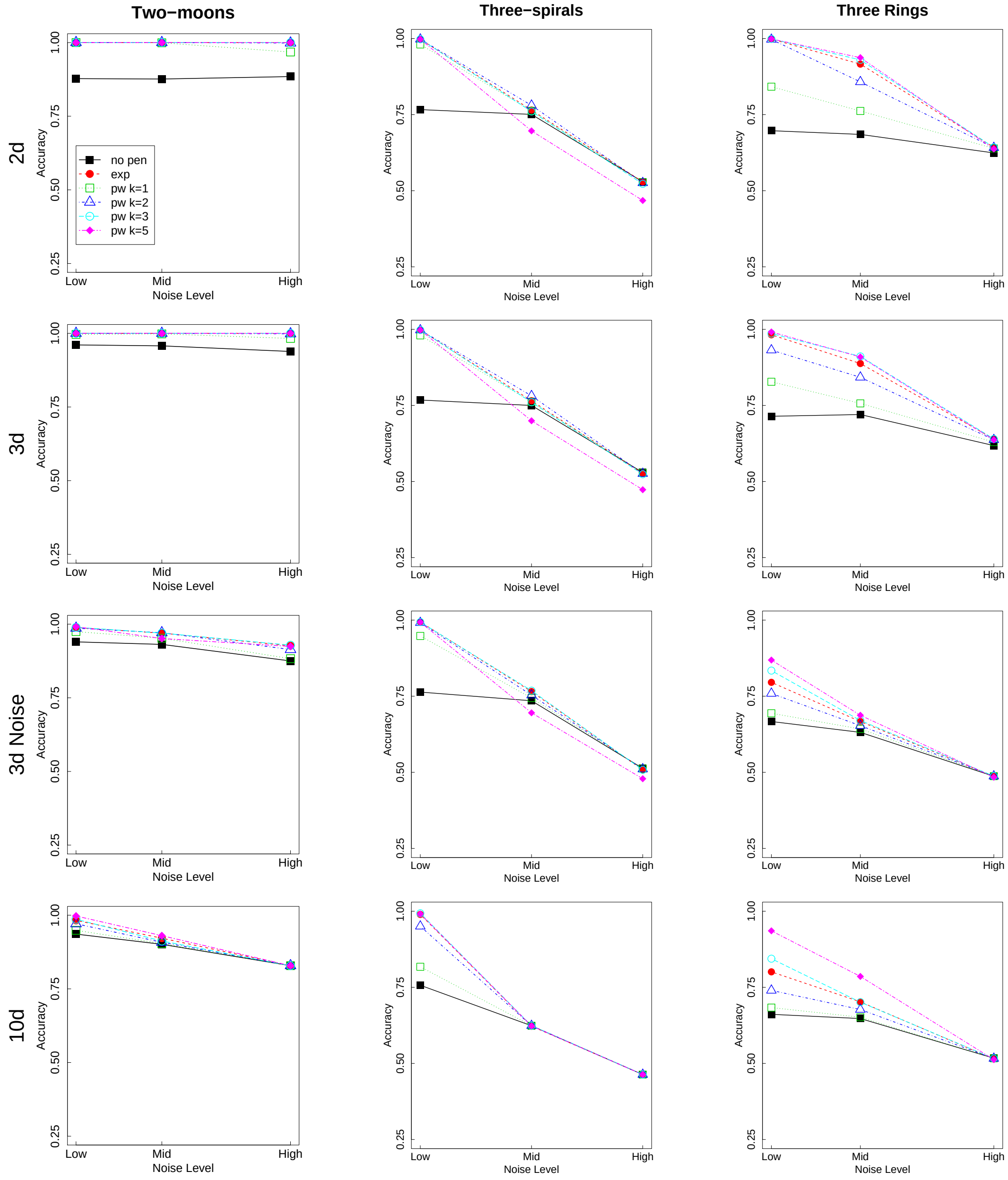

Figure 2: Results of using a Power function penalization $w = d\ (d/\mu)^k$ for four different powers k.

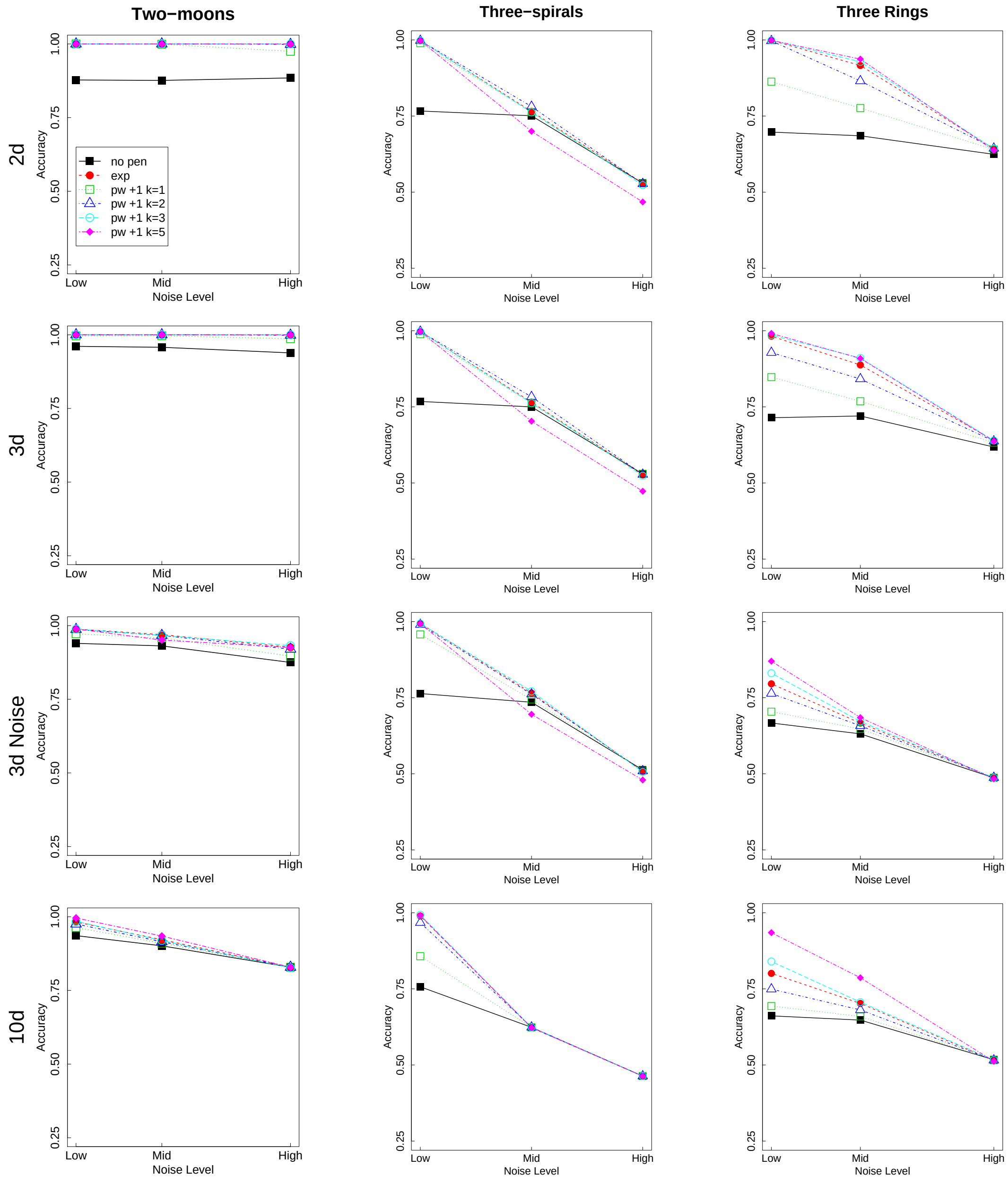

Figure 3: Results of using another power function penalization $w = d\ (d/\mu{+}1)^k$, for four different powers k.

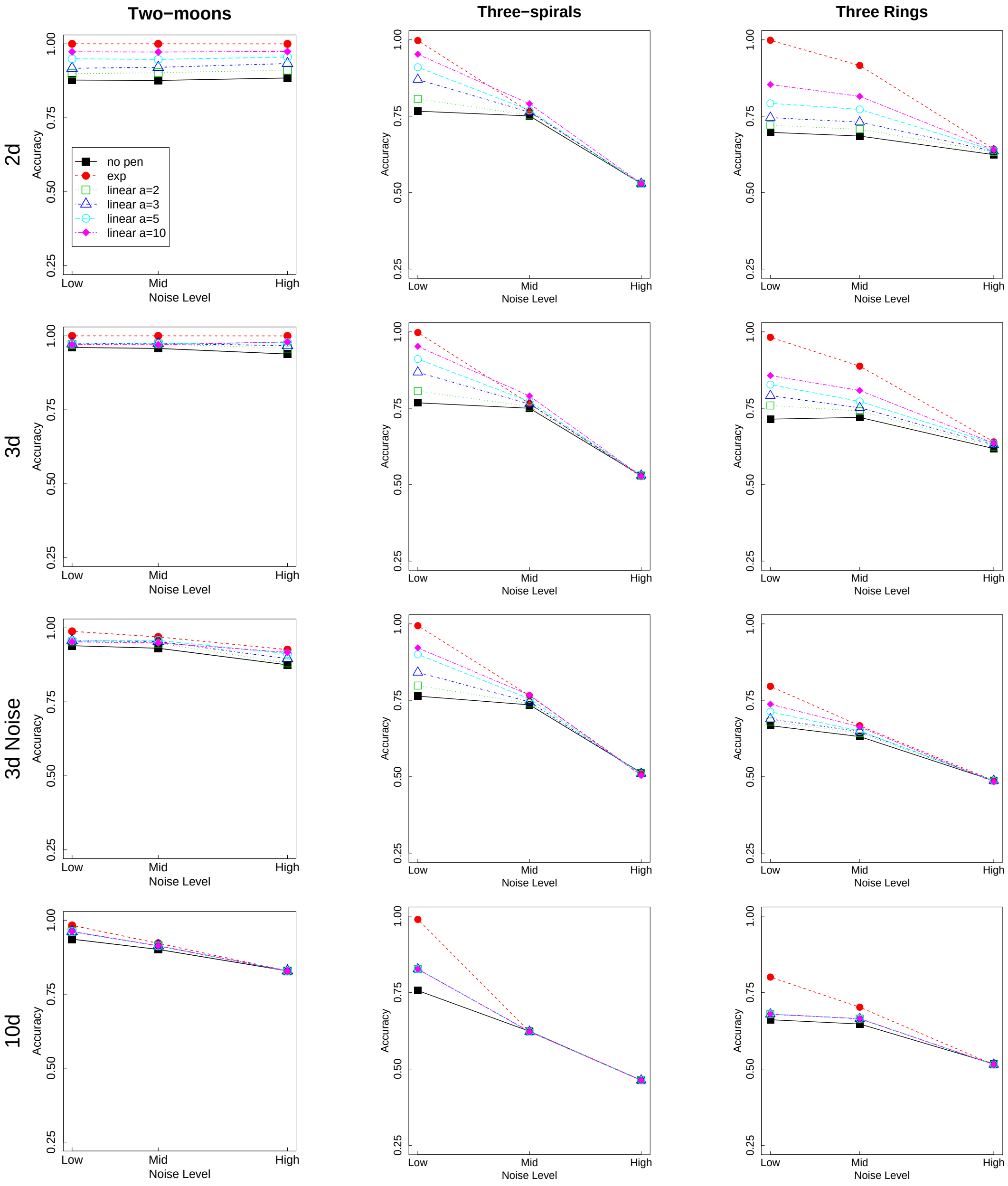

Figure 4: Results of using a linear penalization $w = ad$ for four different a coefficients.

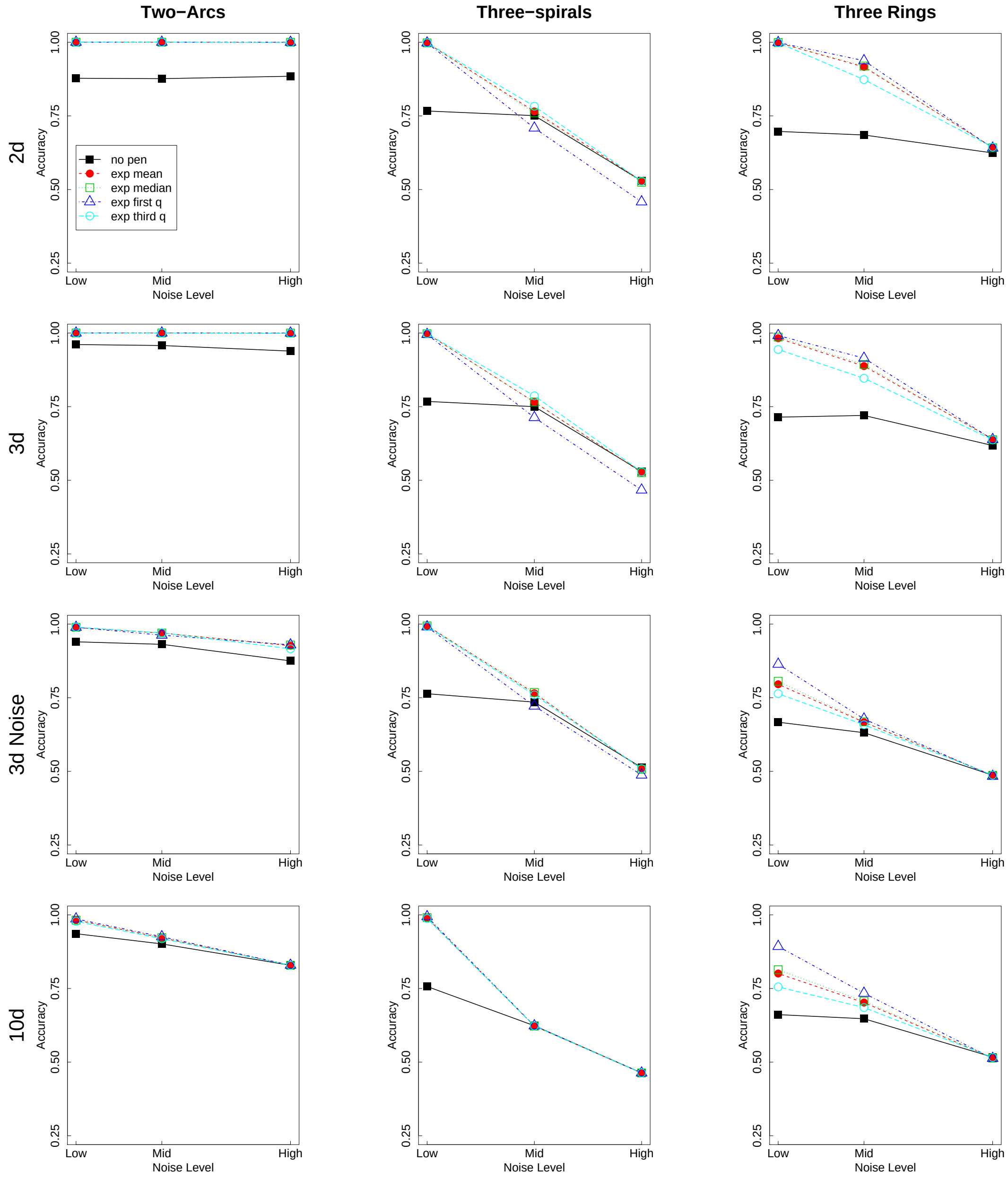

Figure 5: Evaluation of four different definitions of the scaling parameter μ for the exponential penalization function.
